# Functionalization Methods of Starch and Its Derivatives: From Old Limitations to New Possibilities

**DOI:** 10.3390/polym16050597

**Published:** 2024-02-21

**Authors:** Arkadiusz Zarski, Kamila Kapusniak, Sylwia Ptak, Magdalena Rudlicka, Sergiu Coseri, Janusz Kapusniak

**Affiliations:** 1 Faculty of Science and Technology, Jan Dlugosz University in Czestochowa, 13/15 Armii Krajowej Ave., 42-200 Czestochowa, Poland; k.kapusniak@ujd.edu.pl (K.K.); s.ptak@ujd.edu.pl (S.P.); m.rudlicka@ujd.edu.pl (M.R.); j.kapusniak@ujd.edu.pl (J.K.); 2 “Petru Poni” Institute of Macromolecular Chemistry, Romanian Academy, 41 A, Gr. Ghica Voda Alley, 700487 Iasi, Romania; coseris@icmpp.ro

**Keywords:** starch, dextrin, maltodextrin, modification methods, controlled and uncontrolled functionalization, food and non-food application

## Abstract

It has long been known that starch as a raw material is of strategic importance for meeting primarily the nutritional needs of people around the world. Year by year, the demand not only for traditional but also for functional food based on starch and its derivatives is growing. Problems with the availability of petrochemical raw materials, as well as environmental problems with the recycling of post-production waste, make non-food industries also increasingly interested in this biopolymer. Its supporters will point out countless advantages such as wide availability, renewability, and biodegradability. Opponents, in turn, will argue that they will not balance the problems with its processing and storage and poor functional properties. Hence, the race to find new methods to improve starch properties towards multifunctionality is still ongoing. For these reasons, in the presented review, referring to the structure and physicochemical properties of starch, attempts were made to highlight not only the current limitations in its processing but also new possibilities. Attention was paid to progress in the non-selective and selective functionalization of starch to obtain materials with the greatest application potential in the food (resistant starch, dextrins, and maltodextrins) and/or in the non-food industries (hydrophobic and oxidized starch).

## 1. Introduction

The existence of matter is closely related to the presence of polymer compounds in it. Natural polymers, i.e., those found in living organisms, both prokaryotic and eukaryotic, constitute the main building and reserve material. Due to the functions they perform and their strategic importance, it is no wonder that these compounds are commonly found in nature. Biopolymers are therefore obtained from natural, renewable sources, unlike most synthetic polymers produced from a non-renewable source such as petroleum. Moreover, polymers of natural origin are biodegradable and therefore not harmful to the environment. In terms of chemical structure, they mainly include polysaccharides, polypeptides, polynucleotides (nucleic acids), and polyterpenes (rubbers). Over the centuries, humanity has learned how to use these natural resources, developing newer methods and techniques for processing them [1]. Polysaccharides have particularly wide possibilities and application potential. One of them, the most common next to cellulose and chitosan, is starch. Its main sources are primarily cereals—maize, wheat, and rice, as well as potatoes and cassava. Thus, access to starch is practically unlimited all over the world, and the undemanding agricultural cultivation of plants rich in it and simple methods of obtaining and extracting it from plant material make it a relatively cheap polymer [2]. In 2022, the value of the global starch market was estimated at approximately USD 60 billion (134.5 million metric tons), and in the future, it may grow to even USD 90 billion by the end of the third decade (approx. 200 million metric tons) [3]. However, the unwavering interest in starch not only in the food industry but also in other industries results mainly from its functional properties. Analysts predict that the upward trend in the use of this biopolymer will continue in the coming years proportionally in all major industrial sectors. First place (over 60% of the market) is taken by the production of food for humans, both traditional (emulsifiers, thickeners, gelling agents, stabilizers, and antifoaming agents) and functional (e.g., prebiotic), as well as beverages and ethanol. The next places are taken by the production of animal feed, the production of medicines and dietary supplements, and the production of paper, textiles, and glue, as well as the increasingly trendy production of biodegradable packaging as an alternative to plastics. It is also expected that in the coming years the demand for modified starch will significantly increase compared to native starch [4].

**Figure 1:**
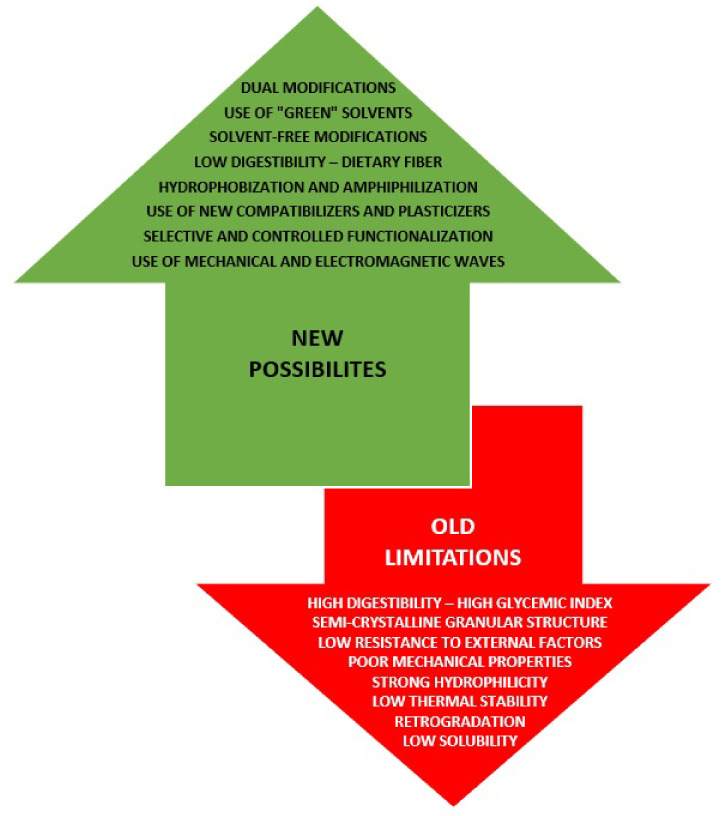
Old limitations and new possibilities in functionalization methods of starch and its derivatives.

In the presented review, the most important issues regarding starch properties that make this biopolymer so attractive for many industrial applications and thereby multifunctional material have been discussed. Special attention was paid to the limitations in its use, as well as to new approaches and strategies proposed or implemented to solve the main problems related to its processing in recent years (Figure 1). The most promising methods and techniques for its further functionalization, as well as the prospects for the development of starch-based materials in the coming decades, have also been described. 

## 2. Structure of Starch

**Figure 2:**
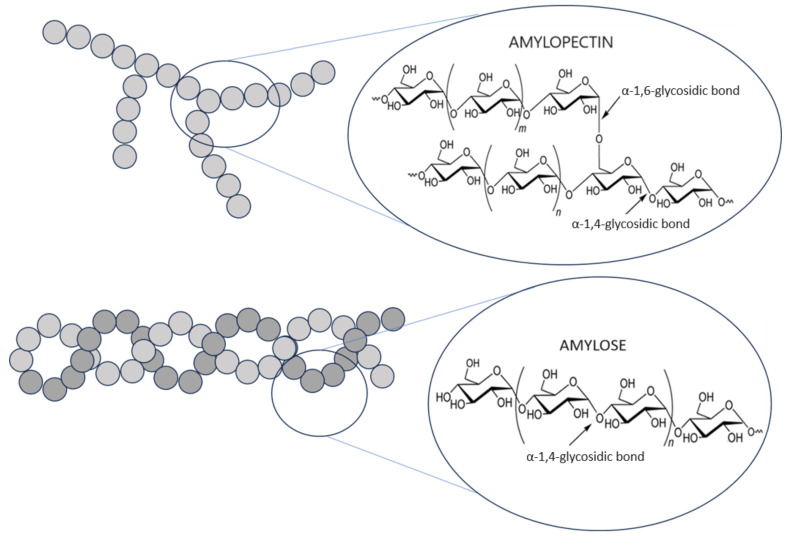
Structure of amylose and amylopectin in starch.

In terms of chemical structure, starch is a polysaccharide. Due to the fact that it consists of only one type of mer, i.e., α-D-glucopyranose, it is classified as a homopolymer. Six-carbon closed rings of glucose units are connected to shorter or longer polysaccharide chains using glycosidic bonds. Such bonds are formed as a result of connecting neighboring molecules through the oxygen atom that forms the hydroxyl group. Chain extension is achieved by replacing the hemiacetal hydroxyl group at carbon C-1 of one glucose molecule with the oxygen atom of the hydroxyl group at carbon C-4 of the next unit, with the release of a water molecule. Therefore, instead of the term glucose unit, the term anhydroglucose unit (AGU) is often used [5]. In this way, one end of the polymer chain, with a free hydroxyl group, is non-reducing, and the other end, with a free aldehyde group, is reducing. In addition to α-1,4-glycosidic bonds, α-1,6-glycosidic bonds also take part in glucose polymerization. While the former are responsible for the creation of chain forms, the latter are responsible for connecting these chains together, resulting in the formation of branched forms. So, basically, starch consists of two polyglucan fractions that differ in structure and, therefore, in properties: linear amylose and branched amylopectin (Figure 2) [6,7]. In amylose, apart from very long polysaccharide chains (average chain length, CL 270–525 AGU), there may be, although definitely few, branches (average CL 5–20 AGU). Amylopectin, in turn, is characterized by many branches but always with short chains [8].

The quantitative ratio of starch fractions is not a constant value and varies significantly depending on the botanical source of starch (Table 1) [6,7,8]. Typically, the main polysaccharide component of starch is amylopectin in a ratio of 3:1 to amylose, with exceptions, such as in the case of waxy starches, where amylopectin constitutes up to 100% of the polymer, or high-amylose starches, as in the case of maize starch with an amylose content of up to 70%. Genetically modified maize starch with an amylose content exceeding 90% is also known in the literature [9,10]. 

The polysaccharide components of starch also differ in molecular weight, which in turn also depends on the origin of the polymer [11,12,13]. Both fractions are identified with the molecular weight distribution throughout the polymer, so that its molecular weight is expressed as the average of the fraction masses. The molecular weight of amylose is much lower compared to that of amylopectin. It ranges from 10^5^ to 10^6^ Da for amylose and 10^7^ to 10^8^ Da for amylopectin [14]. For example, the degree of polymerization (DP) of potato amylose is approx. 10^3^ glucose residues, i.e., the average molecular weight is approx. 162 × 10^3^ Da, while for wheat amylose, it is 4 × 10^3^ glucose residues and 648 × 10^3^ Da, respectively. The number average DP of amylose is between 9 × 10^2^ and 3 × 10^3^ and that of amylopectin is in the range of 5–17 × 10^3^ [9].

In addition to polysaccharide compounds, starch may contain other components, including lipids, proteins, phosphorates, minerals, mainly in the form of oxides, and water—chemically bound or unbound. The content of the latter is very important from the point of view of many properties and applications of starch. In commercially available starches, its content ranges from 12 to 21% (including cereal starches up to 14%, root starches up to 17%, and tuber starches up to 21%). Due to the form in which water may appear in starch grains, it is classified as adsorbed, crystallized, or inclusionary (filling free spaces inside or intermolecularly). Depending on atmospheric conditions (mainly temperature and relative air humidity), its content may change significantly [5,11].

**Figure 3:**
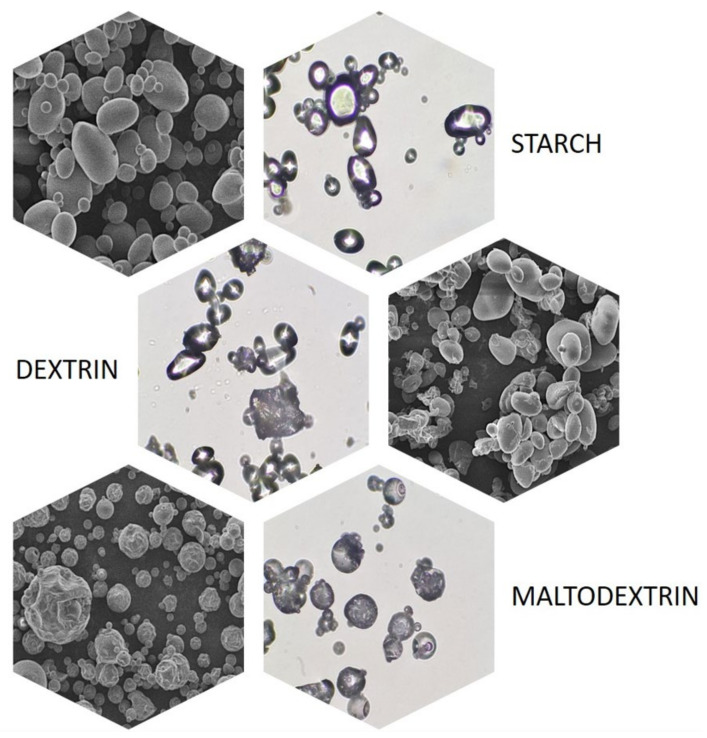
Optical and scanning electron microscope images of potato starch, dextrin, and maltodextrin (Jan Dlugosz University in Czestochowa, 2023).

Both amylose and amylopectin do not occur in nature as separate compounds but in the form of water-insoluble, discrete, and semi-crystalline aggregates called starch granules. These granules, depending on their botanical origin, differ significantly in morphological terms, especially in terms of size (from 1 to 100 μm) and shape (round, oval, spherical, hull, or irregular) [15]. Changes in the appearance of starch granules also occur in the case of most of its modifications, which is particularly well seen in the example of potato starch and its acidically or enzymatically hydrolyzed derivatives—dextrins and maltodextrins, respectively (Figure 3).

Starch granules have a semi-crystalline structure, with a degree of crystallization ranging from 15 to 45%, mainly related to the presence of a branched fraction, i.e., amylopectin. The general model of the crystalline parts of starch granules is based on a spherocrystalline assembly of amylopectin molecules, composed of double helices arranged radially in such a way that the non-reducing end of the polymer chain is directed towards the granule surface. The chains of the branched fraction layer on top of each other in two dimensions, and together with the chains of the unbranched fraction, they organize into three-dimensional micelles, forming radially spreading crystallites with a size of approx. 9 nm [5,16]. It is believed that α-1,6-glycosidic bonds in amylopectin, i.e., branching sites, are located in amorphous areas and, together with amylose, form amorphous fractions of the polymer. Both amylose and amylopectin molecules, through intra- and intermolecular hydrogen and hydrophobic bonds, lead to the formation of structures with an increasingly higher degree of organization—from lamellas, through clusters, blocklets, and growth rings, to the final product, i.e., water-insoluble granules with alternating crystalline and amorphous layers [16].

Various arrangements of double helices are responsible for polymorphism in starch. Polymorphic variants of type A, B, C, and V are known [17]. In type A, amylopectin double helices are short (DP 11–16) and densely packed into monoclinic unit cells with max. eight water molecules. This type is characteristic of cereal starches. In turn, in the type B polymorphic variant, amylopectin helices are long (DP 30–45) and loosely packed with hydration of up to 27%. Type B is often found in starches isolated from tubers and roots. The type C polymorph is a mixture of types A and B. It is characteristic of legume starch. Dehydration caused by high temperature and pressure may lead to changes in the double helical systems and thus polymorphic changes from type B to type A. In turn, type V is characteristic of amylose, fatty acids, and monoacylglycerols. It occurs in swollen granules and becomes visible during gelatinization [18].

## 3. Properties of Starch

Although starch is a homopolymer in terms of structure, it is a highly differentiated compound in terms of its physical and chemical properties. The specific physicochemical properties of this biopolymer depend on many factors, primarily the content of polysaccharide fractions and non-polysaccharide components, as well as the structure and structural features of starch granules. Such differences are dictated not only by genetic conditions—the species origin of the plants from which starch was isolated—but even by the growing conditions of these plants [12].

Starch isolated from plant material after appropriate processing is a white or yellow powder—insoluble in cold water and most common organic solvents. Typically, especially alkaline media used led to its partial hydrolysis and the formation of undesirable depolymerization products. Due to its chemical structure and the presence of many polar hydroxyl groups, it is a hydrophilic compound and should mix well with water, but it does not. It depends on the presence of an insoluble or sparingly soluble amylose fraction. However, starch is hygroscopic—it absorbs large amounts of moisture from the environment. Swelling is a reversible and exothermic process during which starch granules can significantly increase their volume [19]. When the aqueous starch suspension is heated, the starch granules usually swell spherically and crack above 50 °C. Thus, the semi-crystalline structure is lost, and the amylose molecules flowing out from the damaged granules begin to co-create a network that retains water and increases the viscosity of the mixture and electrical conductivity. Gelatinization is often associated with starch pasting but incorrectly so because these terms describe different stages of hydration and swelling of starch granules. It is an endothermic process taking place at a specific temperature or, more precisely, a certain temperature range. Moreover, it is characteristic for a given type of starch and depends not only on the presence of water but also on the pH or presence of ionic compounds, e.g., salt. Gelatinization mainly includes changes related to the melting of crystalline areas of amylopectin and partial washing out of amylose. All the changes taking place around the starch gelatinization temperature are called pasting. During it, intense swelling occurs, and the grains completely disintegrate. In the first phase, hydrogen bonds in the amorphous fraction are broken. In the second phase, water takes on the role of a plasticizer, which results in hydration and the swelling of the mentioned fraction. Ultimately, as a result, the starch loses its granular structure, amylose is intensely released, and the whole thing takes the form of a paste [20].

Equally important parameters as the gelatinization temperature are the reduced viscosity values, which determine the rheological properties of starch, which are particularly important for the food industry. They are used as an indicator of retrogradation during storage. The higher final viscosity and higher degree of retrogradation are caused not only by amylose but also by proteins and the presence of disulfide bridges [21]. If we take a closer look at the retrograde phenomenon, this is an irreversible process based on secondary crystallization. As a result of nonspecific reconstruction of hydrogen bonds between hydroxyl groups, the intermolecular spaces become narrowed, which is accompanied by dehydration called syneresis. The endothermic transformations of retrograded starch may occur at lower temperatures than in the case of native starch. During retrogradation, the crystal lattice is not recreated, but a new one with a lower degree of order is created, which causes the structure to have lower thermal stability. In retrograding starch, amylose associates in a double helix (up to 70 AGU), and amylopectin recrystallization occurs by connecting the outermost and short branches of the polysaccharide chain [22]. 

The functional properties of starch may change over time, under the influence of various biotic and abiotic factors that act directly or indirectly on the degree of granule ordering. The deformation or destruction of the crystalline structure of starch results in mostly irreversible changes in properties, not only corresponding to an increase in amorphousness but also a loss of optical birefringence, gelatinization, and swelling. A high degree of crystallinity ensures structural stability, and thus, the granules become more resistant to various types of phase transitions. Also, differences in the degree of structural order of starch determine its different characteristics in relation to gelatinization temperatures, transition to the glassy state, or decomposition [23].

**Figure 4:**
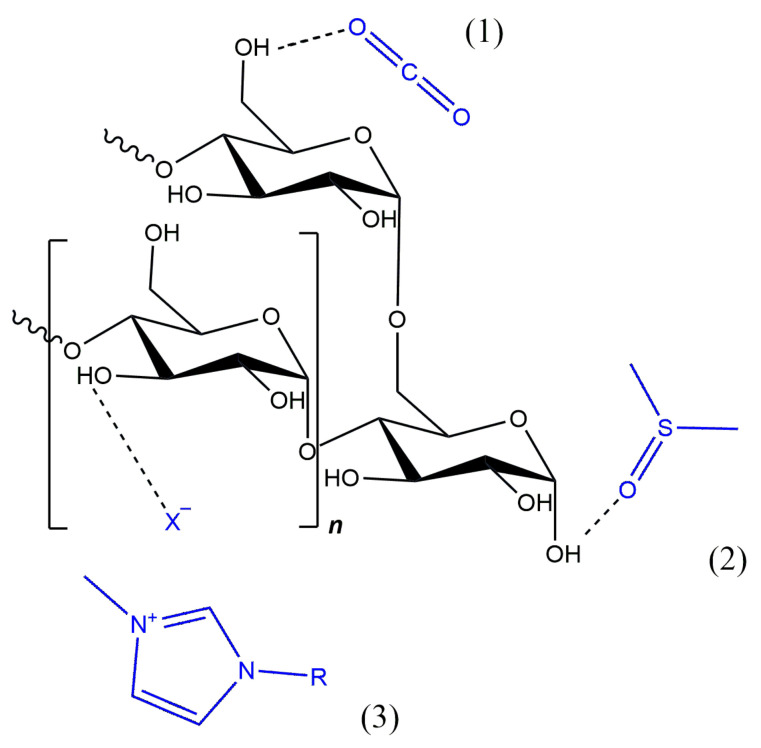
Popular solvents in starch functionalization: (**1**) scCO_2_, (**2**) DMSO, and (**3**) imidazolium IL.

The poor solubility of starch in water and organic solvents is the result of many intra- and intermolecular hydrogen bonds between its hydroxyl groups. When using water as a solvent, the modification is usually carried out in heterogeneous conditions, where we are dealing with a suspension rather than a solution. In a heterogeneous system, starch substitution reactions, for example, yield products with a low degree of modification and many byproducts [24]. Therefore, in order to achieve a higher degree of functionalization, organic solvents and catalysts, such as dimethyl sulfoxide (DMSO), pyridine, N,N-dimethylacetamide (DMAc), or N,N-dimethylformamide (DMF), were quite often used. The problem with most organic solvents is that they can be dangerous to living organisms and the environment due to, for example, their flammability or toxicity. Already for the third decade now attention has been paid to new types of solvents—mainly ionic liquids (ILs) and supercritical fluids (SCFs) (Figure 4) [25]. And it is to them that the following considerations will be devoted.

ILs are compounds with an ionic structure where the cation is only organic, but the anion may also be inorganic. They owe their name to their low melting point, which is below the boiling point of water and often even close to room temperature [26]. Due to their thermal and chemical stability, low volatility, and non-flammability, as well as designable polarity, they are used in various polymerization and derivatization techniques. So far, imidazolium ILs, mainly chlorides, have most often been used in starch modifications. They act not only as solvents but also as catalysts and have usually been used in substitution reactions [27]. In recent years, ionic liquids have been quite often used not only as solvents in chemical and biochemical modifications but also as plasticizers or compatibilizers in physical modifications, mainly to obtain starch blends and TPS [28]. Over the years of their use, some limitations have already arisen. The studies on the ecotoxicity of the ionic liquids known so far have confirmed that they are not as green solvents and reaction media as previously believed [29,30]. The high polarity of compounds used to modify starch, including ionic liquids, is advisable when dissolving or gelatinizing starch but undesirable in anhydrous and biocatalyzed reactions. The hygroscopicity of solvents may create problems with the removal of water as a byproduct of the reaction. This may, in turn, contribute to the inactivation of biocatalysts through the structural deformation of their active centers [31].

A liquid or gas becomes a supercritical fluid (SCF) when the temperature and pressure at which they are located exceed critical values. Of all supercritical fluids, the most commercially available are water (scH_2_O) and carbon dioxide (scCO_2_). The latter has most commonly been used in starch functionalization. For carbon dioxide, the supercritical state occurs above a temperature of 304.25 K and a pressure of 7.39 MPa, in which it retains a density similar to a liquid and a diffusion similar to a gas. Compared to traditional solvents used in starch processing, it is characterized by low viscosity, good permeability, and a high diffusion coefficient, which increase the dissolving capacity, making the mass transfer process simpler and the reaction rate higher. Among the physical modifications of starch, scCO_2_ has been used mainly to induce its gelatinization [32,33]. Chemical modifications of starch using scCO_2_ as a reaction medium include the synthesis of starch esters using methyl and vinyl esters or acetic anhydride, as well as the synthesis of pre-gelatinized starch grafted with poly(L-lactic acid) [34,35,36]. Dual physicochemical methods have also been performed, such as reactive supercritical fluid extrusion, mainly to crosslink starch [37,38,39]. The scCO_2_ has similar advantages to ILs, i.e., non-flammability, non-volatility, and multifunctionality. Additionally, for supercritical fluids, there is no problem with their recycling, as is the case with ILs. The one problem, which is quite significant, is the high cost of producing supercritical fluids. However, when comparing them with ionic liquids, the latter have many more limitations such as the difficulty in their purification and the increasing number of reports about their ecotoxicity [40].

## 4. Physical, Chemical, and Dual Modification of Starch

**Figure 5:**
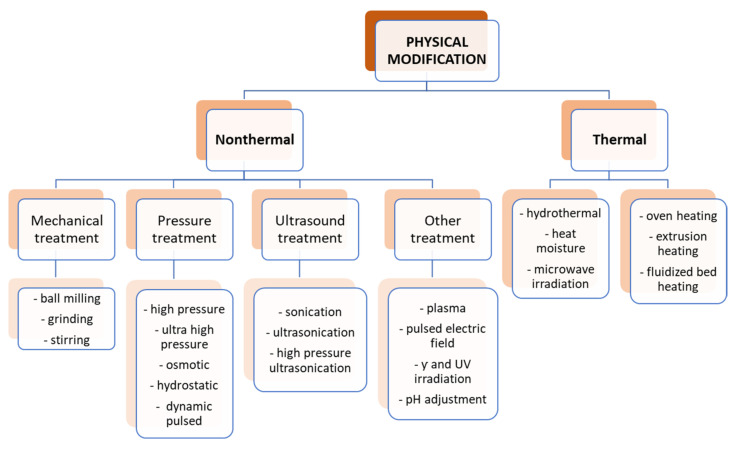
Physical modification of starch.

Starch is a polymer compound that is sensitive to a lot of physical factors such as high temperature, very low and high pH, pressure, light, radiation, ultrasonic waves, and various types of mechanical stress. In general, the physical modifications of starch can be divided into two main groups, i.e., thermal and nonthermal, as shown in Figure 5.

In recent years, nonthermal physical modifications have become increasingly popular. Thanks to them, with low energy consumption, it is possible to introduce significant changes in the structure of starch and thus modulate some of its properties, mainly physical ones. Among them, for example, ozonation forces the loss of starch granulation, and treatment with high hydrostatic pressure affects its crystallinity and gelatinization [41,42]. Nonthermal methods can also induce certain chemical changes, e.g., in the case of plasma, which is able to induce crosslinking in starch molecules, thus introducing changes in the thermal properties of starch—melting temperatures and other phase transitions [43,44].

Among other nonthermal physical modifications, the use of ultrasound and micronization, as well as high-pressure, γ-irradiation, or pulsed electric field treatment, is becoming increasingly popular. Apart from improving the properties of starch, their main advantage is that they are quick and simple and do not require complicated procedures. Moreover, they do not generate byproducts, so the problem of their disposal disappears, and they are not toxic. However, the problem is the high cost of devices and the lack of standardization and reproducibility [45].

When using physical modification, however, as the leading method in dual modifications, hydrothermal methods such as annealing or heat moisture treatment (HMT) were most often used (Table 2).

Annealing uses an excess of water (in the range of 40–70% *v*/*v*) and a temperature below the gelatinization temperature and has a long processing time. HMT is performed at a low water content (usually 10–30% *v*/*v*) and high temperatures of 100–120 °C. In particular the products of combined hydrothermal treatment are promising candidates for resistant starch-based food, being a safer replacement of chemically crosslinked starch [53].

**Figure 6:**
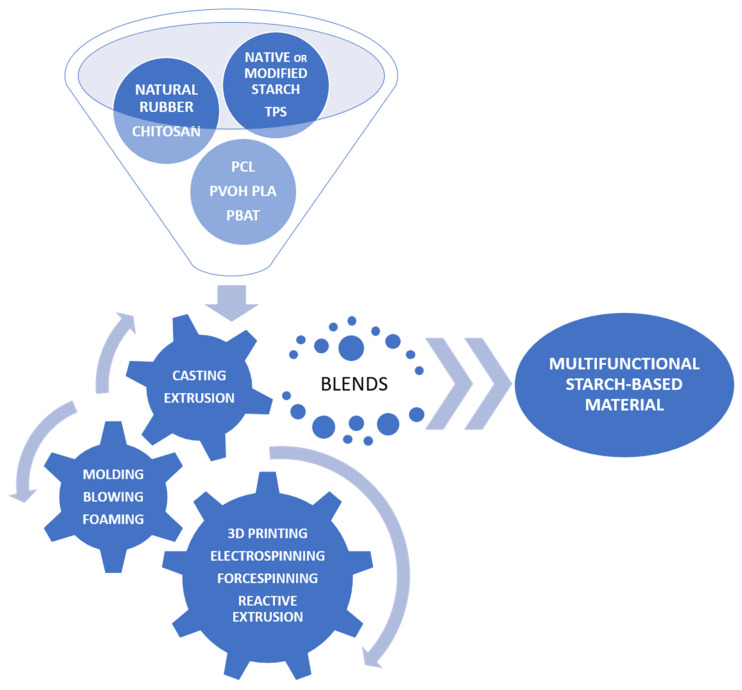
Systems and methods for preparing starch-based blends most frequently used in recent years.

Various methods of preparing blends based on starch or its derivatives are still focused on good compatibility and efficient processing. Such blends have evolved quite dynamically over recent years, from systems with native starch, thermoplastic starch (TPS), or starch modified in various ways to nanostructured varieties. Initially, synthetic, nondegradable polymers were used as blend components. Finally, natural and biodegradable polymers were used. There is an equally wide range of available technologies for their preparation—from typical laboratory or traditional ones such as the solvent casting method, extrusion, molding, and the foaming process to reactive extrusion, 3D printing, or electro- and forcespinning (Figure 6) [54]. A definite trend should be to design materials with improved processing and utility properties while maintaining biodegradability and the nature of environmentally friendly materials.

The combination of starch with synthetic or other natural polymers in the form of blends is carried out in order to eliminate limitations in its use, mainly such as brittleness, sensitivity to moisture, or poor mechanical properties. When a nondegradable polymer is a component of the mixture, there is a risk of greater environmental pollution with microplastics [55]. It is not an ideal solution when we use other biodegradable polymers, such as aliphatic polyesters, in blends with starch. Its strength properties improve, its hydrophilicity decreases, and its resistance to biotic and abiotic factors increases. However, the problem is that these are low-starch mixtures, and their production costs are still high and therefore uncompetitive compared to traditionally used petroleum-based polymers. The evolution in obtaining such materials, opportunities, and challenges was recently reported by Zarski et al. [56] and Muñoz-Gimena et al. [57].

Also, the approach of using starch-based materials in the form of nanostructures is becoming more and more trendy every year [54]. So far, their production has involved both physical processing—grinding, homogenization, ultrasound, and radiation—as well as chemical processing, such as hydrolysis, copolymerization, and crosslinking. It has been shown that the combination of chemical and physical methods makes it possible to achieve greater efficiency and uniformity of the obtained starch nanostructures. Mainly nanocrystals, nanofibers, nanogels, and starch nanomicelles were tested (Table 3). The purpose of producing such nanoparticles is to potentially use them as fillers to improve the barrier and mechanical properties of widely used polymeric materials. 

Nanostructured forms of starch can be used as emulsion stabilizers, additives to films and gels to improve functional properties, forming agents in self-assembling structures, or as scaffold components in tissue engineering. Starch nanoparticles are biocompatible and non-toxic but also sensitive to changes in temperature and acidity. Their properties depend on hydrophobicity, charge, and surface morphology and, above all, size and shape [68].

**Figure 7:**
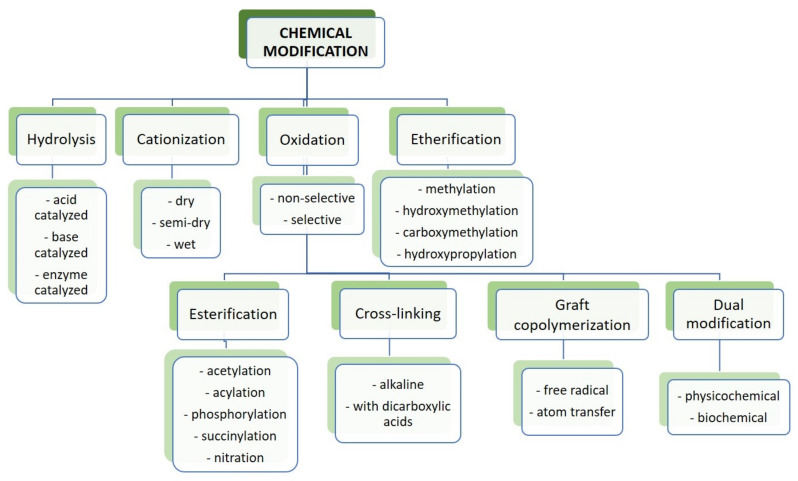
Chemical modification of starch.

**Figure 8:**
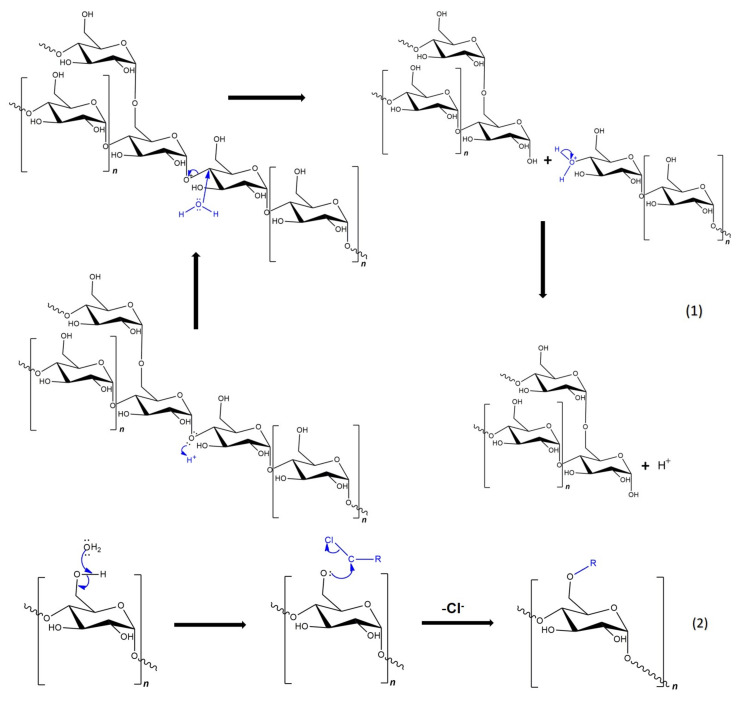

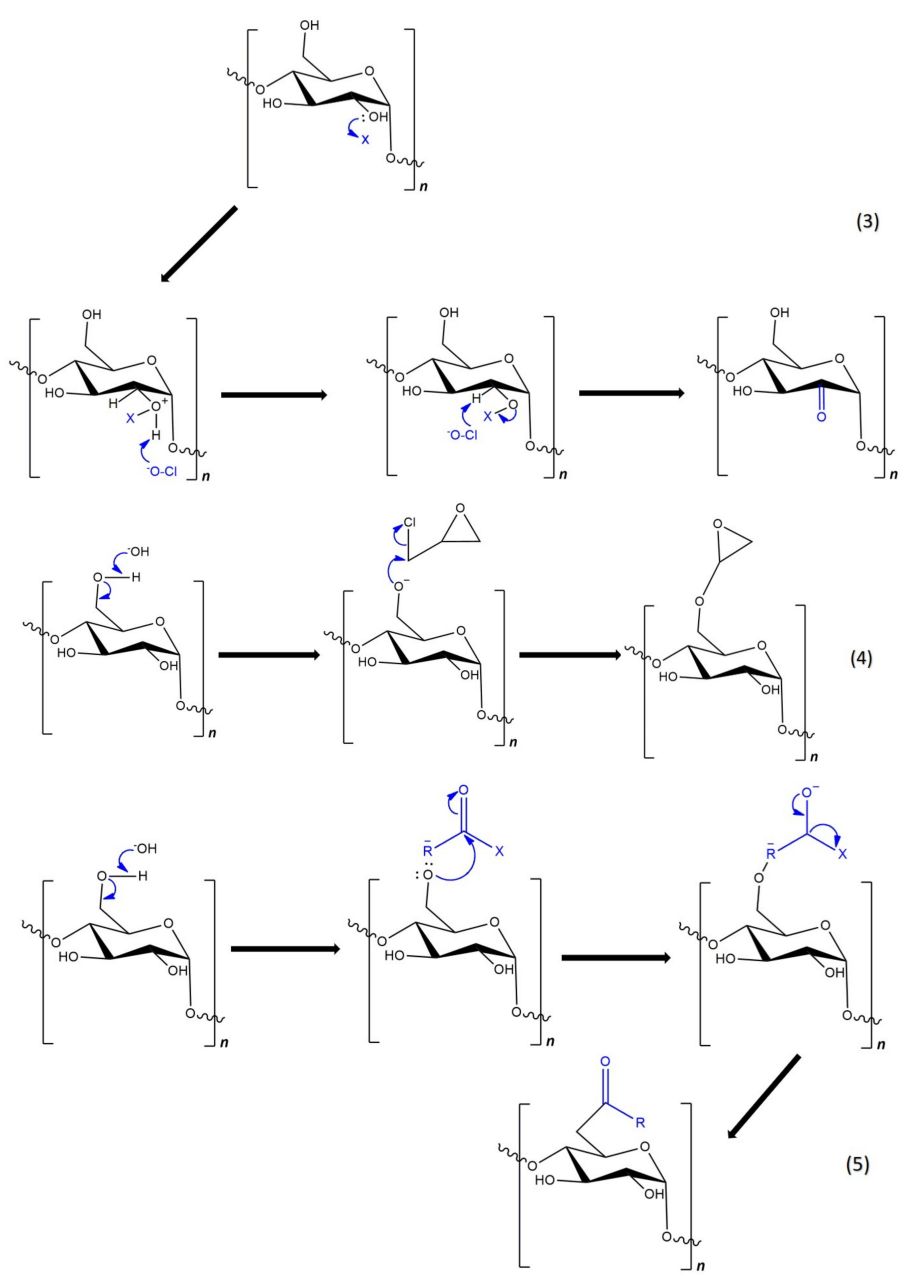
Mechanisms of basic reactions in chemical modification of starch: (**1**) acid-catalyzed hydrolysis; (**2**) etherification; (**3**) oxidation; (**4**) crosslinking; and (**5**) esterification.

Based on the number of studies conducted in recent years, it seems obvious that of all the methods used in starch processing, typical chemical methods are the most important due to the scope of application (Figure 7) [69,70]. The most commonly used chemical functionalization of starch is based on classic organic chemistry reactions such as crosslinking, grafting, esterification, etherification, hydrolysis, or oxidation (Figure 8). 

All these reactions lead to starch derivatives, whose physicochemical properties differ substantially from those of the starting material [71]. Due to its chemical structure, starch has characteristics typical of aldehydes, ethers, and especially alcohols. The ether bond (C-O-C) occurring in the glucopyranose ring of starch is quite stable and not very reactive. It is only susceptible to acid or base hydrolysis at elevated temperatures. The situation is similar with glycosidic bonds responsible for the formation of chains and branches in this polysaccharide. They undergo hydrolysis in the presence of hydrogen ions through the action of strong acid and increased temperature (uncontrolled method)—and hydrolases during enzymatic decomposition (controlled method). The unbranched fraction, i.e., amylose, is more susceptible to hydrolysis. In turn, the glycosidic bonds of both fractions are stable in an alkaline environment, even at elevated temperatures [72]. Therefore, the most susceptible to chemical interactions seem to be hydroxyl groups, of which starch has many, up to three free groups per AGU. These are primary groups at carbon C-6, the most reactive groups, and secondary groups at carbons C-2 and C-3. However, the reactivity of native starch is low due not only to the polymer structure but also to the multi-stage organization of starch granules. Accessibility to hydroxyl groups and glycosidic bonds is hampered by the presence of a dense network of intra- and intermolecular hydrogen bonds [5]. Thus, starch reactions are determined not only by the reactivity of its functional groups and reagents. These are usually reactions at the phase boundary, which, given the complex structure of starch, prevents the effective penetration of the reagent into the potential reaction site. This explains the fact that the degree of starch conversion is usually not great, and a number of functional groups of glucose units remain intact. In turn, those functional groups that are available to the reagents do not always react selectively because local disturbances in the reagent concentration occur. Hence, there is a need not only to develop methods of loosening the starch structure and increasing the potential reaction sites but also to develop selective modifications. Therefore, an effective solution seems to be to conduct regioselective reactions based on one of three approaches: the use of protecting groups, regioselective replacement of -OH groups with other functional groups, or catalyzed selective functionalization of these groups without the use of a protecting group [73,74]. The selective functionalization of starch is discussed later based on the examples of starch oxidation.

Based on current research, it can be concluded that today, single physical treatments or single-step chemical or enzymatic modifications of starch are used less and less frequently. Although it has been emphasized that physical modifications are a much simpler, cheaper, and greener alternative to starch functionalization than in the case of chemical or enzymatic modifications, after many decades of using both, it can be concluded that the truth lies somewhere in the middle because carrying out a more selective or controlled modification requires the use of catalysts, while many chemical or biochemical reactions of starch would not take place without simple or more complicated physical thermal or nonthermal pretreatments such as gelatinization, pH changes, or radiation treatment. Therefore, due to the awareness of the preponderance of potential benefits, especially when designing multifunctional starches, in recent years, more and more attention has been paid to dual or multi-stage modifications.

In the following subsections, we decided to focus on and discuss in more detail recent reports on the non-selective and selective functionalization of starch towards obtaining materials with, in our opinion, the greatest potential in the food industry (resistant starch, dextrins, and maltodextrins) and/or in non-food industries (hydrophobic and oxidized starch). To the best of our knowledge, this is the first review to present such a summary of the studies from the last few years.

### 4.1. Development of New Resistant Starch and Pyrodextrins

Starch is the largest energy source worldwide, but only part of the starch consumed in our diet is degraded by host or bacterial enzymes and absorbed in the form of glucose in the small intestine [75]. Some dietary starch transits the colon as resistant starch (RS), where it is digested by specialized members of the microbiota [76,77]. Moreover, starch can be used as a raw material for the production of preparations with the properties and structure of RS, as well as resistant dextrins (RDex). Both of them may exhibit the characteristics of soluble (SDF) or insoluble dietary fiber (IDF) [78]. RS and RDex as dietary fiber (DF), due to its beneficial health effects, can also be considered as an important ingredient in the formulation of functional foods [79]. Functional foods are industrially processed or natural foods that when regularly consumed within a diverse diet at efficacious levels have potentially positive effects on the health and/or well-being of people beyond basic nutrition [80]. Moreover, some ingredients of DF, including part of RS and RDex, can be selectively utilized by the host microbiota and promote health, demonstrating a prebiotic effect [81]. DF is a functional food ingredient that can be used easily in various functional foods like breakfast cereals, bread, cookies, cakes, yogurt, beverages, and meat [79]. 

#### 4.1.1. Resistant Starch

There are five known types of resistant starch:

- RS1—physically inaccessible starch mainly due to physical barriers formed by cell walls and protein matrices [82];
- RS2—starch that forms compact granules that resist digestive enzymes [83];
- RS3—retrograded starch formed during cooking and the subsequent cooling of starch or starchy products [84];
- RS4—chemically modified starch, i.e., starches which have been etherized, esterified, or cross-bonded with chemicals [85];
- RS5—natural or manufactured starch–lipid complexes [86].

Based on the number of the most cited papers in recent years, more attention has been paid to the impact of the consumption of RS on the gut microbiota and short-chain fatty acid production [76,87,88,89]. However, some scientists are working on the development of new types of RS. 

**Figure 9:**
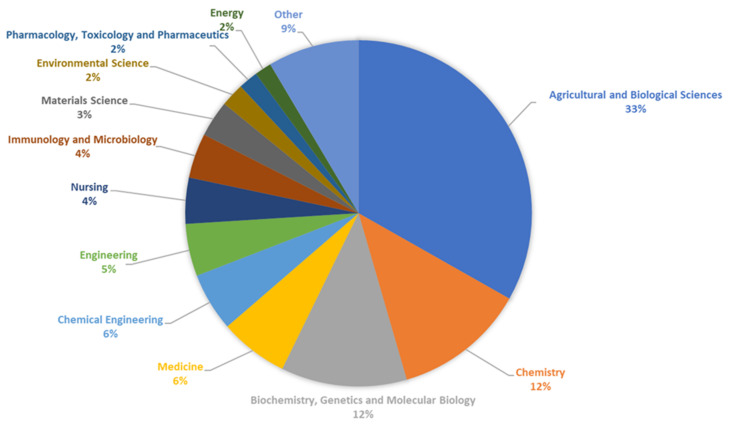
The percentages of documents by subject area on resistant starches published between 2013 and 2023, based on Scopus database.

In recent years, we have observed an increase in the number of publications and a constantly high number of patents devoted to resistant starch. The percentages of documents by subject area on resistant starches published over the last 10 years are presented in Figure 9. 

The progress in the work on their production over the last 10 years is summarized in Table 4. In the case of RS, depending on its type, its resistance may be the result of both its natural structure and the effect of heat/cold or chemical treatment [77].

#### 4.1.2. Pyrodextrins

Pyrodextrins, also called resistant dextrins (RDex) or dextrins (Dex), are produced by roasting dry starch granules with or without acid catalysts [165], whereas resistant maltodextrins (RMDex) are often produced by combined pyrodextrinization and subsequent treatment with amylolytic enzymes [166]. Starch dextrinization causes a series of chemical reactions, including hydrolysis, transglucosidation, and repolymerization [167]. Before Dex were found to be resistant to digestion with amylolytic enzymes, white dextrins, yellow dextrins, and British gums were used as binders, coatings, adhesives, and encapsulating agents [168]. Currently, many studies have confirmed the branched structure of Dex and the formation of new glycosidic bonds (mainly α-1,6, β-1,6, α-1,2, and β-1,2 linkages), ensuring enzymatic resistance [169,170]. To further increase the digestive resistance, sequential application of pyroconversion and enzymatic hydrolysis with alpha-amylase is used [166,171]. RDex/RMDex can be obtained from starches of various botanical origins [172] and are typically amorphous, highly soluble in water, and are characterized by low viscosity and cold storage stability [173,174].

**Figure 10:**
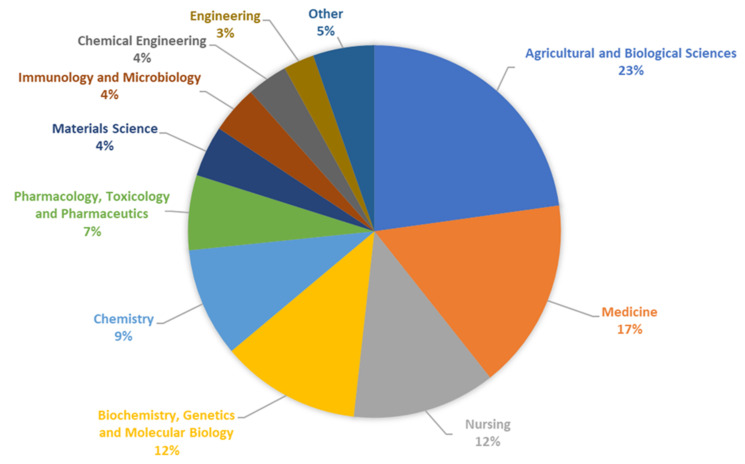
The percentages of documents by subject area on resistant dextrins published between 2013 and 2023, based on Scopus database.

In recent years, we have observed a significant increase in the number of publications and patents devoted to resistant dextrins. The percentages of documents by subject area on resistant dextrin published over the last 10 years are presented in Figure 10.

The progress in the dextrinization of different types of starch over the last 10 years towards DF (and/or prebiotic preparations) is shown in Table 5.

As shown in Table 5, for the purpose of dextrinization, typically acid concentrations of about 0.1% of starch dry basis, heating temperatures ≥ 90 °C, and prolonged heating times ≥ 60 min are applied. The most frequently used catalyst for the dextrinization reaction is hydrochloric acid; however, acids such as acetic, citric, or tartaric acid are also used. Convectional heating is usually used for RDex preparation; however, unconventional methods such as microwave heating can also be successfully used. The latter allows for a reduction in heating time from hours to seconds.

As described above, the resistance to enzymatic digestion of starch derivatives can result from carrying out chemical reactions, including dextrinization reactions. 

### 4.2. Progress in Synthesis of Amphiphilic Derivatives of Maltodextrin

Maltodextrins are the products of partial starch hydrolysis. They are composed of anhydroglucose units, linked together mainly by α-1,4-glycosidic and, less frequently, α-1,6-glycosidic bonds [200]. They are a mixture of molecules with different chain lengths characterized by a common parameter—glucose equivalent (DE) [201]. It is defined as the percentage amount of reducing sugars converted to glucose in the dry weight of the sample [202]. It is inversely proportional to the molar mass and affects the viscosity, sweetness, solubility, and color of maltodextrin [203]. Maltodextrins with different DE values show different properties, such as viscosity [204]. However, also those whose DEs are identical may exhibit different properties. The reasons for this may be different procedures for the preparation of maltodextrins (a type of hydrolysis) or a botanical source of starch [205].

Due to their properties, non-toxicity and a relatively low price, maltodextrins are often used as thickeners in the food industry and binders in the pharmaceutical industry [206,207]. Despite having many advantages, maltodextrins also possess some properties that are unattractive for essential food applications. Due to their highly hydrophilic properties (absence of lipophilic groups), they do not have surface-active properties in emulsion systems [208]. The stabilization of an emulsion with the addition of maltodextrins may be the result of a change in the viscosity of the system or continuous-phase gelling [209]. It is then necessary to use a large amount of maltodextrin, even in the order of 25–40% by weight [200,205].

Such a high content of maltodextrin in food products could significantly change the properties of the final products, not only affecting their viscosity but also the subsequent sensory experience (deterioration of palatability). Moreover, the increase in water activity caused by the use of maltodextrins affects the microbiological stability [210]. An effective solution to this problem could be the use of amphiphilic compounds as emulsifiers.

Amphiphilic polymers have hydrophobic and hydrophilic subregions; therefore, they can act as low-molecular-weight surfactants. They may show good fat emulsifying capacity, possibly due to the steric stabilization associated with their macromolecular structure [211].

**Figure 11:**
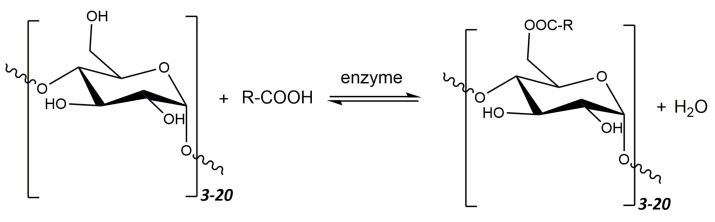
Schematic diagram of the process of converting maltodextrin and fatty acid to maltodextrin ester through esterification reaction using lipase enzyme.

Maltodextrins, in order to change their properties into amphiphilic ones, can undergo various types of modifications—chemical, physical, or enzymatic, e.g., esterification or gelation. The -OH groups at the 2, 3, and 5 carbon atoms are particularly susceptible to reactions. Esterification is currently one of the most popular methods of maltodextrin modification. The general scheme of the esterification reaction is shown in Figure 11.

As a result of esterification, products can be obtained with properties depending on the type of acid used, the glucose equivalent of maltodextrin, and the degree of substitution of the finished product. Additionally, the use of enzymes as esterification catalysts offers an attractive alternative to the synthesis of oligo- and polysaccharide esters. Such reactions can be carried out under milder conditions (lower temperature and pressure), which prevents the depolymerization of polysaccharides [212]. Enzymatic catalysis reduces the need to use toxic reagents (mainly solvents) and can also be carried out under milder pH conditions. These types of reactions are safer for health and may be of interest for biomedical and food applications (emulsifiers, stabilizers, targeted food) [213].

Maltodextrin fatty acid esters are non-ionic surfactants. Due to their amphiphilic character, they exhibit properties different to those of unmodified maltodextrins. They can be obtained as a result of lipase-catalyzed esterification with fatty acids in non-aqueous solvents in the presence of a small amount of water [214]. The mentioned method is of great interest due to the lower amount of byproducts and the mild reaction conditions [215]. Maltodextrins esterified with fatty acids using enzymatic methods have potential applications in the food industry as emulsion stabilizing particles.

Sun et al. [203] obtained maltodextrin esters in a *t*-butyl alcohol environment, using immobilized lipase from *Candida antarctica* as a biocatalyst. In the reaction, they used maltodextrin and stearic acid in a molar ratio from 1:2 to 1:6. They received products with a degree of substitution (DS) ranging from 0.003 to 0.017. The process was carried out at temperatures from 60 to 70 °C for 48–72 h. They considered the conditions as optimal, with the highest DS at a molar ratio of 1:4, a temperature of 60 °C, and a time of 60 h.

Udomrati and Gohtani [207] esterified tapioca maltodextrin (DE = 16) with free fatty acids (decanoic, lauric, and palmitic) in the presence of immobilized lipase from *Thermomyces lanuginosus* in a DMSO environment and with a molar ratio (D-glucose acid/fatty acid) equal to 1:0.1, 1:0.5, and 1:1. In the reaction, they used temperatures in the range of 50–70 °C and incubation times from 2 to 8 h. The obtained products showed a DS of 0.002–0.084 and a solubility in the range of 84.8–87.2%. The highest DS results were obtained for the molar ratio of 1:0.5, at a temperature of 60 °C and after 4 h of the reaction. Studies have also shown that the chain length used in the esterification of the fatty acid affects the degree of substitution obtained during the reaction. This is due to the limited mobility of the longer-chain acids in the reaction system, which in this case leads to compounds with a lower degree of substitution.

The obtained esters were used as emulsifying agents for hexadecane oil. Udomrati and Gohtani [207] investigated their emulsification index as a function of the concentration of maltodextrin/esterified maltodextrin versus oil. They showed that as the concentration of esterified maltodextrins increased, the emulsification index increased, reaching the maximum at a concentration of 35%. The emulsifying activity also increased with the increase in the chain length of the fatty acid present in the ester molecule. Esterified maltodextrin showed surface, interfacial, and emulsifying activity. These results showed that esterified maltodextrins can be used as stabilizers for hydrophobic particles in an aqueous environment. Their absorption at the oil–water interface creates a dense layer of hydrophilic loops, ensuring steric repulsion between the surfaces of the particles [213].

Udomrati and Gohtani [216] expanded their research. They prepared maltodextrin esters, not only based on maltodextrin with DE = 16 but also with DE = 9. They used the esterification method described in [207]. Maltodextrin (with DE = 16 or DE = 9) and fatty acid (decanoic, lauric, or palmitic) in a molar ratio of 1:0.5 were dissolved in DMSO. The reaction was catalyzed by lipase from *Thermomyces lanuginosus* (in a buffer solution). The samples were incubated for 4 h at 60 °C. The obtained esters were tested for the ability to stabilize emulsions containing Tween 80. The emulsions were prepared on the basis of soybean oil, 1% aqueous Tween 80 solution, and aqueous solutions of esterified maltodextrins with various concentrations ranging from 0% to 35%. The results indicated that esters of long-chain maltodextrins with long-chain fatty acids had the best emulsion stabilizing properties at high concentrations. Compared to unmodified maltodextrins, maltodextrin esters proved to be more effective stabilizers for oil-in-water (O/W) emulsions containing Tween 80. They can also be expected to be good emulsion stabilizers of other types.

The continuation of the research described above [217] led to the recognition of the mechanism of emulsion stabilization. The emulsions were prepared on the basis of soybean oil, 1% aqueous solution of Tween 80, and 10% aqueous solutions of esterified maltodextrins (maltodextrin palmitate with DE=16 and maltodextrin palmitate with DE = 9). The described emulsion stabilization mechanism may indicate that mainly Tween 80 has been adsorbed to the oil surface, and maltodextrin esters may interact with Tween 80 to form a double stabilizing layer. The esterified maltodextrin formed an outer dense layer providing steric stabilization between the surface molecules.

Roczkowska et al. [218] carried out the esterification of medium-saccharified potato maltodextrin with oleic acid in the presence of a lipase from *Thermomyces lanuginosus* (in a buffer solution) without the use of any organic solvent. Using the optimal reaction conditions, a molar ratio of maltodextrin to acid of 1:2, a reaction time of 1 h, and a temperature of 60 °C, they obtained a product with a DS equal to 0.024 and reduced the solubility in relation to unmodified maltodextrin.

In a further stage of research on the esterification of potato maltodextrins with oleic acid, a reaction with low- and high-saccharified maltodextrin was also carried out [219]. Products with a DS of 0.038 for the low-saccharified maltodextrin ester and 0.017 for the high-saccharified maltodextrin ester were obtained. On the basis of the obtained esters, O/W emulsions were prepared with various volume ratios of the oil phase to the water phase and with different emulsifier concentrations (10–35%) in the water phase. The emulsions were visually inspected, and the creaming index (CI) was determined. The best results were obtained for low-saccharified maltodextrin ester, 1:1 and 3:2 volume ratios, and emulsifier concentrations in the range of 15–35%. Potato maltodextrin esters with oleic acid can play an important role in the preparation of stable O/W emulsions without the need for an additional stabilizer.

**Figure 12:**
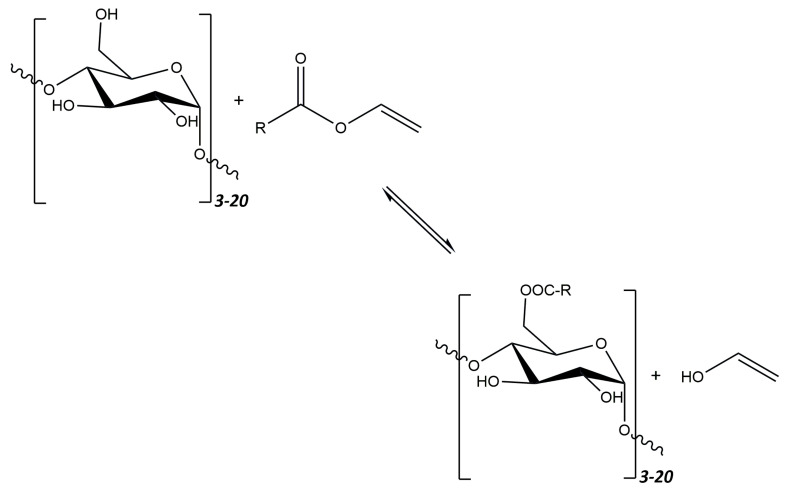
Schematic diagram of the process of converting maltodextrin and vinyl ester to maltodextrin ester through transesterification reaction using lipase enzyme.

One of the newer methods proposed by Park and Walsh [220] is the synthesis of esterified maltodextrins with fatty acids in food-grade ethanol. This distinguishes this method from the previous ones, where solvents such as DMSO or butanol were used. In addition, during the synthesis, two different fatty acid residues were used, both individually and in the form of a mixture. The acid residue donors were vinyl laurate and vinyl palmitate. In the synthesis of esters, corn maltodextrins with two different glucose equivalents, Maltrin 100 (M100) and Maltrin 250 (M250), were used. The reaction was carried out for 8 days in the presence of the immobilized enzyme—lipase from *Thermomyces lanuginosus*. The obtained products were characterized by determining, inter alia, their DS (ranging from 0.016 to 0.026), and solubility (ranging from 93.1% to 100.9%). The general scheme of the transesterification reaction is shown in Figure 12.

It has been shown that the degree of substitution of esterified maltodextrins is closely related to the length of the fatty acid chain used in the reaction. Fatty acids with shorter chains make it possible to obtain derivatives with a higher DS. Esterified maltodextrins formed by a reaction with a fatty acid mixture show an intermediate DS. The solubility of the obtained compounds is not closely related to the DE of maltodextrins but to the type of fatty acid used. Esterified maltodextrins containing shorter-chain acids are more soluble.

On the basis of the obtained products, O/W emulsions were prepared. Corn oil was used as the oil phase. It was mixed in a 1:4 weight ratio with an aqueous solution containing esterified maltodextrins. The derivatives were added to the water in three different concentrations (0.125%, 0.25%, or 0.5%). For comparative purposes, emulsions based on unmodified maltodextrins (specimens M100 and M250), with the addition of sucrose ester, Tween 20, and a blank were also made. Emulsification was carried out at two different temperatures (4 and 22 °C). The emulsions were subjected to stability assessment and droplet size analysis. It was shown that all the modified maltodextrins obtained showed a higher emulsion stabilizing ability than their unmodified counterparts. In particular, emulsions containing derivatives obtained by reacting M100 with vinyl laurate and a mixture of vinyl laurate and palmitate, as well as M250 with vinyl laurate at all tested concentrations and temperatures, showed better results than emulsions prepared on the basis of commercial equivalents. This confirms the validity of the statement that the esterified maltodextrins can be used in the food industry as emulsion stabilizers. The product resulting from the esterification of M100 with 0.5% vinyl laurate at a concentration of 0.5% may prove to be the most effective in this role, especially in the case of food emulsions stored at temperatures from 4 to 22 °C.

An important aspect in food production is microbiological food safety. Limited knowledge of the antimicrobial properties of fatty acid-modified maltodextrins prompted scientists to undertake research in this field. Pantoa et al. [221] investigated the surface-active and antimicrobial properties of three maltodextrin esterification products with fatty acids (in the concentration range from 0 to 20% by weight) obtained on the basis of decanoic, lauric, and palmitic acids. The products were obtained according to the procedure proposed by Udomrati and Gohtani [207]. All products showed surface-active properties that depended on the length of the fatty acid chain and the concentration used. The analysis of antimicrobial properties was carried out on the basis of the ability to inhibit the growth of Gram-negative (*Escherischa coli*) and Gram-positive (*Staphylococcus aureus*) bacteria. The esterified maltodextrins containing the rest of the lauric acid showed the most effective inhibitory effect on the growth of *Escherichia coli* at a concentration of 20%. Unfortunately, none of the esters showed an inhibitory effect on the growth of Gram-positive bacteria.

Subsequent studies on microorganisms by Park and Walsh [222] proved that the esterified maltodextrins obtained by them [220] also have antimicrobial properties. The study used 11 types of microorganisms most often found in food products: yeasts (*Zygosaccharomyces rouxii*, *Zygosaccharomyces bailii*), Gram-positive bacteria (*Bacillus subtilis*, *Streptococcus thermophilus*, *Lactococcus lactis*, *Lactobacillus plantarum*, *Geobacillus stearothermophilus*), Gram-negative bacteria (*Escherischa coli*, *Pseudomonas fluorescens*), and pathogenic Gram-positive bacteria (*Bacillus cereus*, *Listeria monocytogenes*). The antimicrobial activity of esterified maltodextrins was assessed based on the determination of the minimum inhibitory concentration (MIC) and the minimum bactericidal and fungicidal concentration (MBC and MFC) [223]. Extracellular protein concentration tests and SEM analysis were also performed. After analyzing the results, it was shown that the product derived from maltodextrin with a lower DE (M100) and containing acidic residues with shorter chains (lauric) has the highest ability to inhibit the growth of microorganisms among the analyzed esterified maltodextrins. The mentioned product showed very good inhibitory properties against all microorganisms. Gram-positive bacteria were more susceptible to the action of esterified maltodextrins than Gram-negative bacteria and yeasts. Lower DE maltodextrin lauric esters can be used as inhibitors of growth of microorganisms responsible for the spoilage of food sensitive to *B. subtilis*, *L. plantarum*, *Z. bailii*, and *Z. rouxii*.

Another method of hydrophobization of maltodextrins is their acylation with stearic acid/vinyl stearate in the medium of an ionic liquid, 1-butyl-3-methylimidazolium dicyanamide (BMIM[DCA]), which also acts as a solvent and a reaction catalyst [206]. The reaction product, maltodextrin stearate, had a DS of 0.6. The proposed range of applications includes coatings, emulsifiers, dispersants, and nanoparticles.

Ma et al. [224] focused on obtaining maltodextrin derivatives covalently linked with hydroxycinnamic acids, e.g., with vanillic acid, caffeic acid, and ferulic acid. They showed that the grafted maltodextrins produced as a result of esterification had greater thermal stability and viscosity than unmodified maltodextrins. Derivatives containing caffeic acid and ferulic acid had the highest ability to inhibit lipid peroxidation. The introduction of hydroxycinnamic acids to maltodextrins provides the obtained derivative with a new function—antioxidant capacity. For this reason, these compounds can be used as non-toxic antioxidants in the food and pharmaceutical industries.

In another study, Ma et al. [225] obtained cinnamic acid-grafted maltodextrin using two-step esterification. The obtained product was used to prepare the wall material of microcapsules containing purple sweet potato anthocyanins. The microcapsules thus obtained had a spherical shape with visible fractures. During the analysis, the stability of the anthocyanins contained in them was assessed. It was shown that the degradation rate of flavonoids enclosed in capsules with esterified maltodextrin was much lower than in capsules with unmodified maltodextrin. This proves the effectiveness of the derivatives obtained as shell materials which act to increase the stability of the microencapsulated compounds.

So far, there are a limited number of reports on the enzymatic esterification of maltodextrins [203,207,208,216,218,219,220]. The described research focuses on obtaining compounds with beneficial properties, simultaneously acting as effective emulsifiers, non-toxic antioxidants, and food additives, reducing the development of pathogenic microorganisms most often found in food products.

Adequate control of the concentration of esterified maltodextrins in food and cosmetic products can affect the stability of the emulsion. Consequently, it can be implemented for the production of emulsions with different physicochemical properties for various applications [216]. Thanks to properties such as the ability to emulsify and foam, good lubricity, solubility, odorlessness, and non-irritating properties, maltodextrin esters can be used not only in the food industry (ice cream, soups, milk drinks) but also in the cosmetic (toothpaste, lotions, lipsticks) or pharmaceutical (coatings, antioxidants) industries [203].

### 4.3. Functionalization of Starch by Improving Its Hydrophobicity

The following subsection focuses on enzymatic modifications of starch to improve its physicochemical properties [71], which limit its wide application in the food and non-food industries [226,227]. Enzymatically catalyzed modifications, compared to chemically catalyzed ones, are characterized by high selectivity and specificity and lower energy requirements and are more environmentally friendly. Starch modification reactions catalyzed by free or immobilized enzymes include esterification, transesterification, hydrolysis, and grafting with polymers and phenols [71]. The subsection below describes starch modifications using esterification reactions with lipases as biocatalysts (Table 6).

#### 4.3.1. Use of Lipases as Biocatalysts in Starch Esterification Reactions

Xin et al. [228] esterified corn starch with palmitic acid using two methods—in organic solvents (DMF/DMSO) and in a solvent-free system. The reaction was catalyzed enzymatically using Novozym 435 (lipase B from *Candida antarctica* immobilized on a macroporous acrylic resin). In the reaction carried out using the classical method in organic solvents, native starch was used, while in the solvent-free process, native starch was first pretreated in a 9% aqueous NaOH/urea solution. The solution was then neutralized with a 15% HCl solution, and the pretreated starch was precipitated with 95% ethanol. A solvent-free system using pretreated starch gave starch palmitate with a degree of substitution (DS) of 1.04, while the DMF/DMSO solvent method gave an ester with a much lower degree of substitution of 0.0072. The obtained starch esters were characterized by a high ability to emulsify and stabilize emulsions due to their hydrophobic properties. Two years later, the authors [227] confirmed that Novozym 435 is the best enzyme to catalyze the esterification reaction of corn starch with palmitic acid, comparing it to immobilized thermophilic fungal lipase (TLIM), *Candida cylindracea* lipase (CRL), and porcine pancreas lipase (PPL). Novozyme 435 had the highest activity compared to the other lipases tested.

The same enzyme (Novozym 435) was used by Lin et al. [229] in the esterification of tapioca starch with rosin acids. The process was carried out with the use of a solvent (DMSO) under various conditions (time, temperature, amount of enzyme) in order to optimize them. A molar ratio of starch to acid of 1:2 was used, and the starch was pretreated similarly to that in the study by Xin et al. [228]. The degree of starch substitution was calculated according to the methodology described by Varavinit et al. [230] and by Lu et al. [231], and it amounted to a maximum of 0.106. The reaction was confirmed with FTIR analysis and SEM images [229]. A year later, Lu et al. [232] carried out the esterification process using the same reagents, enzyme, and solvent. Starch was also pretreated with the same method as in the previous work [229]. A molar ratio of starch to acid of 1:1 was used, and the degree of substitution was a maximum of 0.092. The reaction was confirmed with FTIR analysis and SEM images. X-ray analysis (XRD) confirmed that new crystal regions were obtained and the original crystal structure was destroyed. The obtained starch esters were characterized by high hydrophobicity, which was confirmed by decreasing solubility in water and swelling power with an increasing degree of substitution (DS). The swelling power of esterified starch with the highest DS (0.092) was about 71% lower than that of native starch. This phenomenon is related to the lower ability of esterified starch to form hydrogen bonds. In addition to improved hydrophobicity, the resulting starch esters were characterized by improved viscosity and emulsifying properties [232].

The enzyme-catalyzed esterification of waxy corn starch with octenylsuccinic anhydride (OSA) was carried out in 2012 by Xu et al. [233]. Novozym 435 lipase was used as the catalyst, and the starch was pre-heated in water (35%, *w*/*w*) at 65 °C for 15 min. Then, lipase and OSA were added in amounts of 0.6% and 3%, respectively. The reaction was carried out for 30 min at 45 °C after adjusting the pH to 8.0. A small band corresponding to the vibration of ester carbonyl groups (1713 cm^−1^) confirmed that OSA starch was obtained. The degree of substitution was 0.0195. The use of a lipase biocatalyst made it possible to significantly shorten the duration of the starch esterification process, which gives it a chance to be used on a larger scale in industry. The resulting OSA starch gelled in a shorter time to achieve higher viscosities at higher concentrations. The starch crystal structure was not changed, which was confirmed with SEM photos and XRD analysis.

The esterification reaction of waxy corn starch with octenylsuccinic anhydride (OSA) catalyzed enzymatically (Novozym 435) was also carried out by Lu et al. [234] in 2016. The process used starch pretreated enzymatically for 4h (temp. 55 °C, pH 5.2) with β-amylase (300 U/g dry weight of starch) and transglucosidase (50 U/g dry weight of starch). Comparatively, the esterification process was also carried out on untreated native starch. The reaction was carried out with 1% or 3% OSA by weight of starch at a pH of 8.5 at 35 °C for 8 h and then stopped by lowering the pH to 6.5. The product was precipitated with ethanol. The highest degree of substitution was obtained for starch pretreated with enzyme and 3% OSA and was 0.0197.

Gao et al. [235] attempted to esterify corn starch with lauric acid, without using any organic solvent. Lipase extracted from porcine pancreas was used as a biocatalyst. The reaction was performed in a small amount of water at 60 °C for 24 h at pH 6.0. The weight ratio of starch to acid was 1:0.5, and the amount of enzyme used was 1% of the dry weight of starch. In the FTIR spectra of the obtained product, no bands characteristic for carbonyl groups were found; only a reduction in bands corresponding to hydroxyl groups in the starch molecule was observed. The degree of substitution was determined with titration, and it reached the maximum of 0.0151.

An alternative to organic solvents, in which the starch esterification reaction is usually carried out, are so-called “green solvents”, such as ionic liquids. The esterification reaction of high-amylose corn starch with methyl palmitate in the presence of lipase from *Candida antarctica* was carried out by Lu et al. [231]. The dried starch was pre-dissolved in a mixture of ionic liquids (1-butyl-3-methyl-imidazolium acetic, [BMIM]Ac, and 1-butyl-3-methyl-imidazolium tetrafluoroborate, [BMIM]BF_4_) and heated at 120 °C for 2 h. Then, the esterification reaction was carried out under various conditions of time (1, 2, 3, 4 h), temperature (50, 60, 70, 80 °C), amount of enzyme (0, 0.05, 0.10, 0.15, 0.20), and the molar ratio of methyl palmitate per anhydroglucose unit (1:1, 2:1, 3:1, 4:1). Based on the XRD analysis and SEM images, it was found that the crystal structure of the starch was destroyed. The modified starch was characterized by increased hydrophobicity compared to native starch, which is hydrophilic. This was confirmed by measuring the water contact angle. Based on the thermogravimetric analysis, it was found that starch esters are characterized by lower thermal stability compared to native starch. The degree of starch substitution was calculated on the basis of the results of the titration analysis according to the methodology described by Varavinit et al. [230]. The maximum degree of starch substitution with palmitic acid that was obtained was 0.144 [231].

The esterification reaction of starch with unsaturated fatty acid in an ionic liquid medium was also carried out by Zarski et al. [236]. Potato starch was pre-gelatinized in an ionic liquid (1-butyl-3-methylimidazolium chloride, [BMIM]Cl), and then the same liquid was used as a reaction medium for esterification with oleic acid. The process was catalyzed by immobilized lipase from *Thermomyces lanuginosus*. The highest DS was obtained in the reaction carried out at 60 °C for 4 h and was 0.22.

Three years later, Zarski et al. [237] again used [BMIM]Cl and immobilized lipase from *Thermomyces lanuginosus* to esterify potato starch. Hydrolysates of high-oleic vegetable oils (pure and waste rapeseed oil) were used as esterifying agents. A small amount of the surfactant polyoxyethylene sorbitan monooleate (Polysorbate 80^®^; P80) was used in the esterification process, which contributed to obtaining starch esters with a very high DS, which reached a value of 1.36 under the most optimal conditions. This also gave the possibility of conducting the reaction at lower and more optimal temperatures for lipases, i.e., 40–50 °C. In addition to physicochemical studies for the resulting starch esters, functional analyses were also carried out [238]. The extruded films were characterized by increased hydrophobicity and better mechanical properties, while maintaining biodegradability and non-phytotoxicity.

The study shows that the use of lipase (immobilized and non-immobilized) as a catalyst for the starch esterification reaction allows the process to be carried out under mild conditions with the reduced use of organic solvents, which can be replaced by so-called “green solvents” [71].

**Table 6:** Esterified starches with lipases as biocatalysts.

Type of Starch	Esterifying Agent	Enzyme	Solvent	Pretreatment	DS Max	Ref.
Corn starch	Palmitic acid	Novozym 435 (lipase B from *Candida antarctica*)	DMF/DMSO	-	0.0072	[228]
Corn starch	Palmitic acid	Novozym 435	Solvent-free system	9% aqueous NaOH/urea solution	1.04	[228]
Tapioca starch	Rosin acid	Novozym 435	DMSO	Aqueous NaOH/urea solution	0.106	[229]
Tapioca starch	Rosin acid	Novozym 435	DMSO	Aqueous NaOH/urea solution	0.092	[232]
Waxy corn starch	OSA—octenyl succinic anhydride	Novozym 435	Water	Heating in distilled water (20–45%, *w*/*w*)	0.0195	[233]
Waxy corn starch	OSA—octenyl succinic anhydride	Novozym 435	Water	Enzymatically treated native starch (β-amylase (300 U/g dry weight of starch) and transglucosidase (50 U/g dry weight of starch)	0.0197	[234]
Corn starch	Lauric acid	Lipase extracted from porcine pancreas	water	-	0.0151	[235]
High-amylose maize starch	Methyl palmitate	*Candida rugosa* lipase (E.C. 3.1.1.3.) (nominal activity: 739 U/mg enzyme) from *Candida rugosa*, type VII	Ionic liquids: 1-Butyl-3-methyl-imidazolium acetic ([BMIM]Ac) and 1-butyl-3-methyl-imidazolium tetraflouroborate ([BMIM] BF_4_)	Dissolution of dried starch in IL mixtures (1-butyl-3-methyl-imidazolium acetic ([BMIM]Ac) and 1-butyl-3-methyl-imidazolium tetraflouroborate ([BMIM]BF_4_)) and heating for 2 h at 120 °C	0.144	[231]
Potato starch	Oleic acid	Immobilized lipase from *Thermomyces lanuginosus*	1-butyl-3-methylimidazolium chloride ([BMIM]Cl)	Pre-gelatinization in 1-butyl-3-methylimidazolium chloride ([BMIM]Cl)	0.22	[236]
Potato starch	Hydrolysates of high-oleic vegetable oils (pure and waste rapeseed oil)	Immobilized lipase from *Thermomyces lanuginosus*	1-butyl-3-methylimidazolium chloride ([BMIM]Cl)	Pre-gelatinization in 1-butyl-3-methylimidazolium chloride ([BMIM]Cl)	1.36	[237]

#### 4.3.2. Use of Microwave Radiation in Starch Esterification Reactions

An additional improvement in the starch esterification process with acids, in addition to catalyzing the reaction with lipases, can be carried out in solvent-free conditions with the use of microwave heating (Table 7). This eliminates the need for organic solvents altogether, allowing for shorter process times while obtaining high degrees of starch substitution [239]. Chemical modifications of starch using methods such as carboxymethylation, acetylation, methylation, and hydroxypropylation result in a low degree of substitution, while requiring a longer reaction time compared to microwave-assisted methods. Microwave heating destroys the crystal structure of starch and increases the contact area between reagents and starch particles [240]. Microwave heating was found to increase the degree of substitution (DS) of starch [241,242,243,244].

Lu et al. [239] attempted to esterify corn starches with different amylose and amylopectin contents using maleic (solid state) and acetic acid anhydride (liquid state). The process was carried out with the use of microwave radiation power of 350 W and different heating times (1, 1.5, and 2 min), which were equivalent to high reaction temperatures of 92, 98, and 108 °C, respectively. The process was not enzymatically catalyzed. The esterification reaction was confirmed with FTIR analysis, XRD, and SEM images. A DSC study was also performed. It was found that the DS of maleic anhydride-modified starches was higher for starch with a higher amylose content (max. 0.274). This is due to the greater amount of hydroxyl groups in amylose, which results in higher microwave sensitivity. At the same time, the DS of acetic anhydride-modified starches was higher for starches with a higher amylopectin content (max. 0.197). This is due to the liquid state of acetic acid, which is thus able to diffuse into the starch granules with a high amylopectin content.

The microwave-assisted esterification reaction of corn starch was conducted in 2021 by Hu et al. [245]. In the process, native starch and pretreated starch with pullulanase were used. The esterifying agent was citric acid. The reaction was carried out under different conditions of temperature and time. It was shown that the samples obtained by combining enzymatic debranching and microwave-assisted citric acid esterification had an altered crystal structure, and a new band appeared in the FTIR spectra, proving the existence of an ester bond. Esterification also affected in vitro enzymatic digestibility, so it can be a method for obtaining resistant starch with high thermal stability.

In 2010, Horchani et al. [246] confirmed that the use of microwave radiation has a beneficial effect on the efficiency of the esterification process. In their study, they used maize starch, oleic acid, and non-commercial CaCO_3_-immobilized lipase from *Staphylococcus aureus* (SAL3). It was proved that the highest degree of substitution of starch with oleic acid (2.86) was obtained after a 4 h reaction carried out first in a microwave field and then in a liquid state in a flask by shaking. The reaction yield was 76%. For comparison, the reaction was carried out under identical optimal conditions using only microwave radiation and only by shaking in the flask. In both cases, lower results were obtained, which amounted to 50% (DS = 1.8) and 45% (DS = 1.6) of the esterification reaction efficiency. In order to optimize the reaction conditions, the molar ratio of starch to acid, the amount of enzyme used, and the reaction temperature were changed. The optimal conditions were a molar ratio of starch/oleic acid of 0.18, 386IU of lipase, a temperature of 44 °C, and a time of 4 h. The obtained product was subjected to physicochemical analysis (FTIR, NMR, viscosity measurement, digestibility, thermal analysis).

Similar studies were carried out in 2016 by Adak et al. [247], who esterified corn starch with oleic acid using lipase from *Rhizopus oryzae*. The process was first carried out in a home microwave oven using different radiation powers (50%, 80%, and 100%) and then in a flask by shaking at 100 rpm at 30 °C for 2, 4, 6, or 8 h. Various enzyme activities were also used (100 IU, 150 IU, 200 IU, and 300 IU). Starch with oleic acid was mixed at various molar ratios of oleic acid/anhydrous glucose units (AGUs) in starch (1:1, 2:1, 3:1, and 4:1). As in the study of Horchani et al. [246], comparative reactions were carried out—only under microwave radiation and only by shaking in a flask to check the reaction efficiency. Additionally, novel ionic liquid-type imidazolium cationic surfactants ([C_16_MIM]Br, [C_16_-3-C_16_IM]Br_2_, and [C_16_-12-C_16_IM]Br_2_) were used. The highest degree of substitution of starch with oleic acid equal to 2.75 was obtained using [C_16_-3-C_16_IM]Br_2_ as a surfactant. A molar ratio of starch to oleic acid of 1:3, 150 IU of enzyme, and heating in a microwave field at 80% for 1 min, followed by shaking the reaction mixture at 30 °C for 4h, were established as the most optimal reaction conditions. The formation of ester bonds was confirmed with FTIR spectra, and changes in crystallinity and structure were verified with XRD analysis and SEM images. The esters of starch and oleic acid showed high water resistance and high thermoplasticity [247].

The esterification of corn starch was also undertaken by Lukasiewicz et al. [248]. Acetic acid, lauric acid, and stearic acid were used as acyl donors. The reaction was carried out with the use of microwave radiation of various strengths and organic solvents (DMSO and DMF). The biocatalyst was commercial hog pancreas lipase (Sigma-Aldrich, Saint Louis, MO, USA) with an activity of 15–35 units/mg. The highest degree of substitution (0.513) was obtained using DMF as a solvent and lauric acid as an acyl donor. A physicochemical analysis proved that these conditions resulted in the lowest degradation of starch and preservation of its granular structure. The use of microwave radiation contributed to reducing the reaction time by about 2.5 times.

Microwave radiation can be used not only during the starch esterification reaction but also for its pretreatment. Zhao et al. [242] performed the acetylation of potato starch with acetic anhydride and glacial acetic acid in a volume ratio of 1:1. The microwave pretreated starch esters showed a higher degree of substitution (0.073) and a lower relative crystallinity compared to the esters obtained from starch not pretreated with microwave radiation.

The use of microwave radiation and enzymatic catalysis in starch modifications gives the opportunity to conduct the reaction in a short time and in solvent-free conditions, which contributes to its lack of negative environmental impact and low costs. The starch esters obtained in this way can be used in various industries—both in food and packaging, depending on the degree of starch substitution obtained. Modifications of the starch-based film leading to ester groups reduce the migration of plasticizers from packaging materials to food, which affects the high safety of their use [249]. 

**Table 7:** Starches esterified using microwave radiation as an energy source.

Type of Starch	Esterifying Agent	Enzyme	Solvent	Pretreatment	DS Max	Ref.
Corn starches with different amylose/amylopectin ratios	Maleic (solid) and acetic (liquid) anhydrides	-	-	-	0.274	[239]
Corn starch	Citric acid	-	Distilled water	Enzymatic debranching of starch using pullulanase	-	[245]
Maize starch	Oleic acid	Non-commercial CaCO_3_-immobilized lipase from Staphylococcus aureus (SAL3)	Solvent free	Heating in distilled water (20–45%, *w*/*w*)	2.86	[246]
Corn starch	Oleic acid	Lipase of *Rhizopus oryzae* NRRL 3562	Phosphate buffer (10 mM, pH 7); novel ionic liquid-type imidazolium cationic surfactants ((C_16_MIM)Br_2_, [C_16_-3-C_16_IM]Br_2_, and [C_16_-12-C_16_IM]Br_2_)	-	2.75	[247]
Maize starch	Acetic acid, lauric acid, and stearic acid	Commercial hog pancreas lipase (Sigma–Aldrich) with activity of 15–35 units/mg	DMSO, DMF	-	0.514	[248]
Potato starch	The acetic anhydride and glacial acetic acid (1:1 in volume)	-	Deionized water	Microwave pretreated	0.073	[242]

### 4.4. Recent Developments in Selective Starch Oxidation

**Figure 13:**
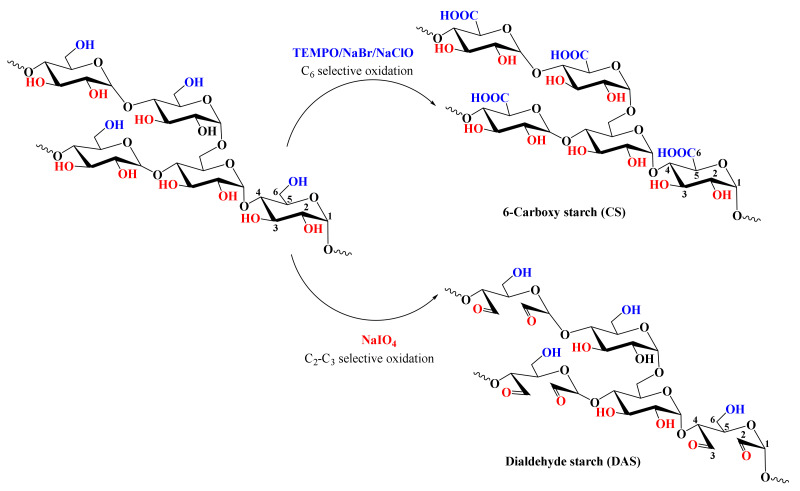
Illustration of starch oxidation employing two of the most selective protocols, TEMPO/NaBr/NaClO and sodium periodate.

Among the most common uses of oxidized starch, due to its specific properties, such as low gelatinization temperature, high fluidity, reduced viscosity, and good transparency, the food industry plays a particularly important role [250,251]. There are many protocols reported in the literature for starch oxidation, which mainly aim at the oxidation of hydroxyl groups bound to C_2_, C_3_, and C_6_ atoms. These methods differ greatly in the nature of the reagents used, regioselectivity, yields, reaction times, pH, etc. Two of the most selective and used oxidation methods are shown schematically in Figure 13. By using a stable nitroxyl radical, i.e., 2,2,6,6-tetramethylpiperidine-1-oxyl (TEMPO), as a mediator for oxidation with sodium hypochlorite (NaClO) and sodium bromide (NaBr) it is possible to preferentially oxidize only the primary OH groups in the starch structure. This protocol is the mainstream, especially for drug delivery applications [252]. Due to its high selectivity, this method is widely used for the oxidation of many other polysaccharides, such as cellulose [253,254,255,256], chitosan [257], or pullulan [258,259]. It is worth mentioning that the oxidation with the TMPO radical takes place in two stages, by means of aldehyde groups, with these being finally converted to carboxylic groups. It is worth noting that the hydroxyl groups linked to C_2_ and C_3_ are not affected by TEMPO-mediated oxidation. Thus, both the breaking of glycosidic bonds and the destruction of pyranose rings is impeded, while preserving the geometry of the original starch polymer. In contrast to this protocol, selective oxidation with sodium periodate (NaIO_4_) occurs at the C_2_ and C_3_ atoms (secondary hydroxyl groups), converting them into aldehyde groups, simultaneously with the cleavage of the C2 and C3 atoms (Figure 13). In addition to these two methods, there are many variants of them, as well as other reagents including hydrogen peroxide (H_2_O_2_) or ozone (O_3_).

#### 4.4.1. TEMPO-Mediated Oxidation of Starch

**Figure 14:**
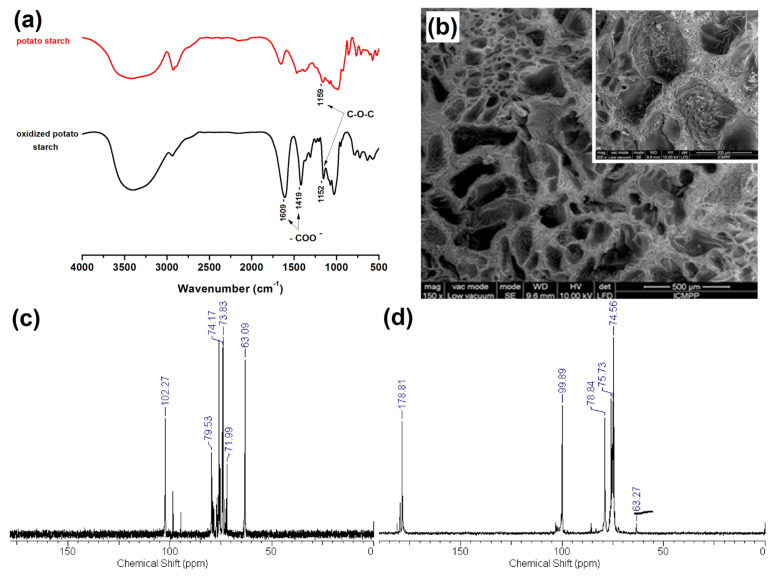
FT-IR spectra of the native potato starch and its TEMPO-oxidized correspondent (**a**); SEM images of semi-interpenetrating polymer network cryogels by crosslinking polymerization of N,N-dimethylaminoethyl methacrylate (DMAEM) in the presence of oxidized potato starch: mag 150× (inset 500×), the scaling bar 500 µm (inset 200 µm) (**b**); and ^13^C-NMR spectra of unoxidized (**c**) and oxidized potato starch (**d**). Adapted with permission from ref. no. [252]; 2016, Elsevier.

As a possible application of the oxidized starch, obtained after a selective oxidation with TEMPO, in one of the recent works, two varieties of starch, i.e., corn and potato, were tested. These were subjected to selective oxidation with the TEMPO radical and NaClO, in order to introduce COOH groups, uniformly distributed along the starch chain, using them for the crosslinking polymerization of N,N-dimethylaminoethyl methacrylate (DMAEM), thus resulting in interpenetrated semi-interpenetrating polymer network (semi-IPN) cryogels, at a low temperature of −18 °C (Figure 14). 

It was assumed that the nature of the starch will have a different influence on the fabricated composite cryogels; more precisely, the altered porosity will induce a different response to external stimuli, such as pH and temperature, but also the content of carboxylic groups will greatly affect the release profile of a model drug (indomethacin) entrapped in the hydrogels [252]. After TEMPO oxidation (Figure 14), in the FTIR spectra of native and oxidized potato starch, the main differences consist of the appearance of the new double sharp peaks at 1609 cm^−1^ and 1419 cm^−1^, revealing the asymmetrical and symmetrical stretching vibrations of COO^−^ groups, formed after the selective oxidation. The selectivity of the reaction is proved by the bell-shaped peak centered at 3420 cm^−1^, assigned to the OH groups in starch, which are still detectable in the oxidized samples, indicating that the secondary OH groups are unaffected by the oxidation. Valuable information was extracted from the ^13^C NMR spectra of the pristine and oxidized potato starch samples. The pristine sample shows peaks of C_6_ at 63 ppm, C_1_ at 102 ppm, and those of C_2_, C_3_, C_4_, and C_5_ in the range of 72 and 79 ppm. After oxidation, the disappearance of the peak from 63 ppm is almost total, concomitant with the apparition of a new and strong peak at 178 ppm, ascribed to the newly formed COOH group. It should be mentioned that no evidence of the ketone group formation (as a potential oxidation of secondary OH groups) was detected, since there was an absence of any chemical shifts in the range of 185–205 ppm (Figure 14c,d). The semi-IPN prepared cryogels are homogeneous with well-defined pores with values of the average diameter of about 50 µm (Figure 14c).

Another TEMPO derivative, i.e., 4-acetamide-TEMPO, has been employed in the selective oxidation of water-soluble starch, pre-dissolved in sodium acetate (0.2 M) solution at 85 °C, with NaClO_2_ as an actual oxidant and NaClO used for triggering the reaction. The oxidation reaction efficiency of the proposed system was tested by using three different temperatures, 4, 25, and 50 °C, for 24 h [260]. The authors observed no significant differences between the oxidized products at 4 and 25 °C, but their major degradation was noticed in the reaction at 50 °C. The catalyst, 4-acetamide-TEMPO, is the essential parameter for achieving high yields with high selectivity. The results obtained in this study revealed that the proposed oxidative system ensures high degrees of oxidation of starch but at the same time leads to the depolymerization processes of products, which is identified with molecular mass analysis performed using size-exclusion chromatography–multiple angle laser scattering (SEC-MALS). Although the reaction has a good selectivity (C_6_), when a longer time is adopted, it was possible to observe the initiation of some non-selective oxidation processes, which occurred in the other OH groups in the AGU in the following order: C_3_ > C_2_ > C_1_ [260]. An ingenious breakthrough for improving the efficiency of TEMPO oxidation on starch has recently been reported [261]. In this report, it has been shown that cellulose nanocrystals greatly improve the oxidation efficiency of the TEMPO/NaClO system. Is believed that nanocrystalline cellulose has the ability to migrate into starch microparticles, thus facilitating their oxidation. The optimum concentration of nanocrystalline cellulose to reach the maximum degree of oxidation was only 0.5% (wt.), with the films thus prepared showing a high degree of transparency (0.66) and a contact angle of 102^o^. Compared to the control and oxidized starch samples (oxidized with TEMPO, without nanocrystalline cellulose), the oxidized starch based on cellulose nanocrystals showed a much-improved hydrophobicity. In conclusion, the cellulose nanocrystals behave as a prooxidant agent and confer clearly superior mechanical properties to oxidized starch samples [261]. 

#### 4.4.2. Dialdehyde Starch Preparation by Using Sodium Periodate

**Figure 15:**
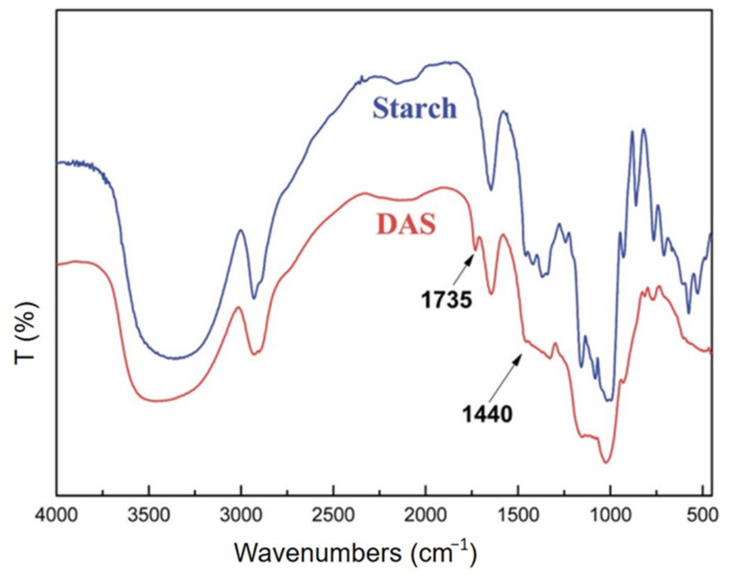
FTIR spectra of corn starch and dialdehyde corn starch. Reprinted with permission from ref. no. [263]; 2015, RSC Publishing.

Through an oxidation reaction to the C_2_ and C_3_ of the anhydroglycoside unit, dialdehyde starch (DAS) is obtained. Because the reaction takes place in secondary OH groups, they are converted to aldehyde groups, while breaking the bond between C_2_ and C_3_ atoms. The reaction product formed after these transformations contains a considerable number of aldehyde groups, which induce new physical properties, such as a much-improved solubility in an alkaline environment and excellent adhesion, being easy to gelatinize, biodegradable, and well tolerated by living organisms. In addition, by introducing aldehyde groups into the structure, broad premises are created for subsequent chemical processes. The first attempts to obtain DAS used a one-step oxidation process, with the oxidizing agent being iodine, which was oxidized to periodate, but the reaction took place with very low yields [262]. Subsequently, separate processes were adopted, with the reaction currently taking place both on a laboratory and industrial scale, in the presence of sodium periodate. Unfortunately, even this system does not provide a significant improvement over the issues previously encountered: yields and reproducibility are still low, especially when using starch varieties with different origins, and it requires large amounts of oxidizing agent and long reaction times. Thus, a lot of effort is involved in identifying ways to optimize this protocol. In such an attempt, an oxidation process has recently been reported which combines two reactions simultaneously: acid hydrolysis and oxidation with sodium periodate. Thus, the authors reported a decrease in the reaction time to only two hours, given that the content of aldehyde groups determined by titration was almost 93% [262]. In another report on this matter, sodium periodate solution was used (100 mL, 0.3 mol L^−1^) under vigorous mechanical stirring to oxidize 10 g of corn starch; the pH value of the solution was adjusted to 1.5 using a hydrochloric acid solution (1 mol L^−1^). The reaction took place at 35 °C for 4 h, avoiding light access [263]. The oxidized product (dialdehyde starch, DAS) was characterized with FTIR spectroscopy (Figure 15).

The cleavage of the C_2_–C_3_ bond of the anhydroglucose unit results in the formation of two aldehyde groups per unit, forming 2,3-dialdehyde starch. The band at 1735 cm^−1^ is the characteristic peak for C=O vibrations, specific for the aldehyde groups, while the weaker peak at 1160 cm^−1^ is assigned to the C–O bond stretching of the OH groups linked to carbon, in the glucose units. Other broad bands in the range of 1360 to 1440 cm^−1^ are characteristic for the C=O symmetric stretching vibrations of carboxyl groups. These findings suggest that both the aldehyde group and carboxyl groups were attached into the structural units by the periodate selective oxidation of the starch. DAS was then used to reduce graphene oxide (GO) and polyaniline (PANI) in one pot to fabricate DAS-reduced graphene oxide (RGO)/PANI composite, which displays good electrochemical properties. Moreover, the maximum specific capacitance of the fabricated composite achieved 499 F g^−1^, at a current density of 0.5 A g^−1^, representing the highest value for graphene-doped PANI composite reported so far, using a green, environmentally friendly protocol for graphene oxide reduction. The designed supercapacitor expressed several other prominent features, such as low resistance, expanded cycle life, and quick reflection of oxidation/reduction on high current changes [264]. One of the most common uses of dialdehyde starch is as a crosslinking agent of different polymeric components for the formation of interpenetrated networks, hydrogels, films, etc., as a replacement for rationally used low molecular aldehydes, such as glutaraldehyde, which are toxic and difficult to purify and remove after synthesis [263]. 

#### 4.4.3. Hydroxyl Peroxide (H_2_O_2_) for Starch Oxidation

Hydroxyl peroxide (H_2_O_2_) has a broad potential to be used for oxidation reactions because of its reduced cost, also being considered as a benign and environmentally friendly compound, safe to use, and providing non-toxic byproducts (i.e., water) [265,266]. Because the oxidation of starch with sodium hypochlorite (sometimes calcium hypochlorite) leads to the formation of chlorine-based residues, for use in the food industry, however, starch oxidation methods are preferred to avoid chlorinated products. In a recent report, oxidized starch used as a stabilizer and fat-reducing additive in dietetic mayonnaise was prepared using a tandem based on a H_2_O_2_ and sodium bicarbonate (NaHCO_3_) redox system in which sodium bicarbonate (NaHCO_3_) played the role of an activator, widely used in medicine, food, and pharmacy, being safe for health. The molar ratio of NaHCO_3_/H_2_O_2_ was 0.2 [267]. Under these conditions, the resulting oxidized starch had much-improved properties, like solubility, emulsifying abilities, swelling, and clarity, as compared with pristine starch, but the crystallinity degree of the resulting oxidized products significantly decreased [267]. Corn starch was oxidized with H_2_O_2_ in a Fenton-like protocol favored by ball milling [267]. The authors achieved the highest degree of carboxyl and carbonyl substitution in the oxidized product, up to 0.950 and 0.573, respectively. They found that the optimized parameters for maximizing the substitution degree are a water content of 20% (based on starch dry basis), a 0.2 molar ratio of H_2_O_2_/anhydroglucose units, a molar ratio of Cu^2+^ to anhydroglucose units of 4.2:1000 (molar ratio), pH 5, and a 6 h reaction time [268]. This new type of oxidized starch, with such a high number of functional groups and low viscosity, is especially recommended for the warp sizing of high-density textile fibers to meet the needs of advanced high-pressure sizing processes, high consistency and small viscosity. Moreover, its use can be expanded towards environmentally friendly and safe materials in the food industry. Another study on starch oxidation using hydrogen peroxide revealed that by varying the H_2_O_2_ concentration, different degrees of oxidation can be achieved, which can be deduced from the continuous decrease in the intrinsic viscosity of the oxidized samples [269].

In general, the oxidation reaction is favored by the temperature, with heating in the microwave system presenting indisputable advantages compared to classic heating: speed, mass uniformity, and cleanliness. Protocols for the oxidation of starch using H_2_O_2_ under a microwave field have already been reported in the literature [266]. Maache-Rezzoug et al. [270] demonstrated that the instantaneous controlled treatment of pressure drops affects the structural and physicochemical features of starch. Bahrani et al. [271] observed that sudden decompression by vacuum treatment with standard maize starch made the granule structure brittle. In a recent paper, the preparation of three types of oxidized starch (corn, cassava, and canna) using a vacuum and H_2_O_2_ as an oxidizing agent has been reported [272]. The oxidized samples at atmospheric pressure were compared with those oxidized in a vacuum, concluding categorically the advantage of using a vacuum as a method for the pretreatment of starch. The authors explain the superiority of the vacuum method, hypothesizing that when starch is subjected to a vacuum, the pressure inside the starch granules becomes higher than the outside pressure, leading to the expansion of the starch granules and their structure becoming brittle; accordingly, the oxidant molecules reach the starch granules more easily. Therefore, this method is particularly efficient, economical, and environmentally friendly [272].

#### 4.4.4. Physical and Chemical Combined Methods for Starch Oxidation

**Figure 16:**
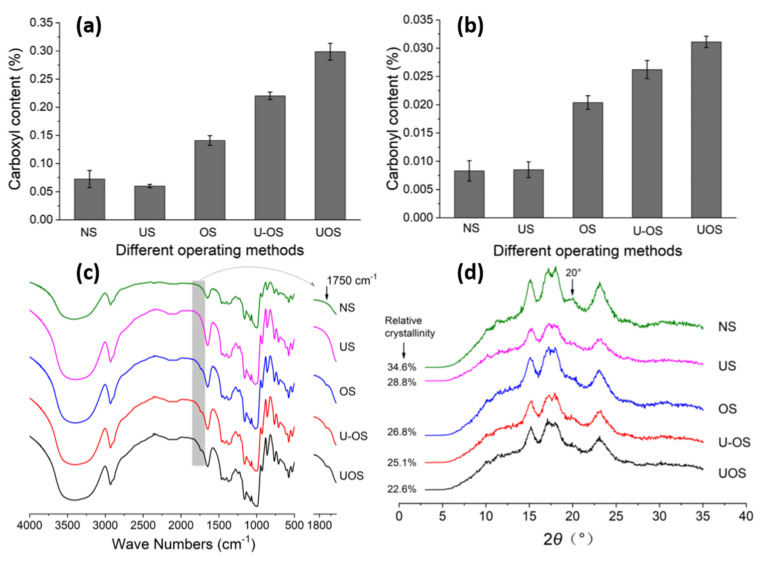
The carboxyl (**a**) and carbonyl (**b**) contents, FTIR (**c**), and relative crystallinity (**d**) of native corn starch (NS) and starch samples studied by means of various treatments: ultrasound-prepared corn starch (US), oxidized corn starch (OS), starch prepared by ultrasonication, followed by oxidation sequentially (U-OS), and starch prepared by both ultrasonication and oxidation (UOS). Adapted with permission from ref. no. [273]; 2019, Elsevier.

A combination of physical and chemical treatments was recently adopted in order to evaluate the differences between the ex situ (ultrasonication of starch and oxidation sequentially, U-OS) and in situ (ultrasonication starch and oxidation simultaneously, UOS) oxidation of corn starch assisted by ultrasonic treatment, studied against traditional oxidized starch (OS), Figure 16 [273].

In this case, the chemical oxidant was NaClO (3% active chlorine) without the addition of any co-oxidant (like TEMPO or NaBr). The reaction was carried out at an alkaline pH (pH = 9.5) for 20 min, and hydrochloric acid was used for the pH adjustment [273]. It can be seen from Figure 16 that the carboxyl content is much improved for the protocols involving ultrasound treatment, with this amount increasing by 56% and more than 110% for the U-OS and UOS samples, respectively, in comparison with the sample oxidized without any ultrasound treatment (sample OS). This behavior suggests that ultrasonic irradiation is a process that can strongly activate the oxidation process while shortening the reaction time. A plausible explanation for this effect would be that ultrasound promotes larger access to the intimate areas of the particles due to cracks and pores formed on the surface [274]. The carbonyl amount formed is much lower than the carboxyl one, see Figure 16, as expected in this kind of reaction, when sodium hypochlorite is used under alkaline media. The oxidation reaction products were easily detectable with FTIR spectra, where the occurrence of a new absorption characteristic peak at 1750 cm^−1^ validates the success of the reaction. In addition, this band has different intensities, which are correlated with the content of carboxylic groups formed, with the increase being in the following order: OS, U-OS, and UOS. However, this protocol also has a major drawback: following the oxidation and ultrasonic processes, the crystallinity of the resulting products decreases significantly (Figure 16d). Thus, the crystallinity of the starting material, located around 36%, decreases to approximately 25% for the U-OS sample and to 22% for the UOS sample. However, this trend is not new, as ultrasound and/or oxidation are likely to reduce the starch crystallinity, as the lattice ordering is somewhat affected [275].

#### 4.4.5. Electrochemical Methods for Starch Oxidation

In recent years, the study of new methods of oxidizing starch without the use of chemical reagents has taken place. In this case, the oxidation is performed by using electrochemical methods [276]. These methods are benign, having a minor impact on the environment, with a number of advantages, such as that [277] the reaction rate can be strictly controlled and the degree of oxidation can be determined at any time by measuring the conductivity of the solution. Additionally, it is possible to interrupt the reaction by turning off the electricity, and the impact of pollutants on the environment is minor; thus, this process is environmentally friendly and economic [278,279]. In a study of the oxidation of starch in the absence of any chemical reagents, particularly valuable information was identified. In this study, UV irradiation and thermal treatment, as two environmentally friendly treatment techniques, were applied for the modification of oat and barley starches, and their effects on different starch properties were juxtaposed with those noticed for starch oxidation using a classical protocol involving NaClO [280]. A decrease in both the molecular weight and the degree of crystallinity of the barley starch was noticed because of the prolonged UV and thermal treatment. Exploring the phenomenon in more detail, through the EPR technique, it was highlighted that the generation of free radicals in barley starch is much more pronounced than in the other type of starch, which explains its lower structural stability. The structural changes in the two types of starch correlated very well with the changes in their physical properties. It was concluded that UV irradiation could be assimilated with a highly effective oxidizing agent, comparable even with the classical chemical reagent (NaClO), while the heat treatment is not as effective, producing mainly undesired depolymerization phenomena of starch chains [280]. Ozone is considered an environmentally friendly alternative for the preparation of oxidized starch especially when its use takes place in the food industry. It is common in the literature, in recent years, having been intensively applied to several varieties of starch [281,282]. The most important parameters that affect the oxidation process are the concentration of ozone and the concentration of the starch suspensions. Unlike other traditional oxidation processes, ozone oxidation occurs through a different reaction mechanism, leading to the predominant formation of aldehyde groups to the detriment of carboxylic groups, leading to particular properties of the products, such as high amylose content, superior capacity swelling, better solubility, clarity of the paste, and lower viscosity than the native ones.

## 5. Summary and Future Perspectives

This review was intended to present the importance of starch, materials based on it and its derivatives, and directions for their development. Attention was paid to the possibilities but also limitations and problems related to its processing. The most popular and most important (top and trendy) methods of starch functionalization for both food and non-food applications were presented. Currently, many methods and various conditions for their implementation are used to functionalize starch, including mechanical and electromagnetic waves. Everything is aimed at the broadly understood optimization of starch processing so as to obtain a product with precisely defined parameters. The unquestionable advantage of starch is its availability and biodegradability. It is quite a challenge to search for the most efficient solutions, with little material and equipment input and additionally without a negative impact on the environment. Since a natural polymer is used, it is worth carrying out all treatments related to its processing in accordance with the principles of green chemistry and sustainable development. The production of food based on both traditional and functional starch, mainly health promoting, is a priority and strategic in the economic dimension. Due to this fact, all non-food applications of this biopolymer are often met with social criticism, and in some countries, they are not recommended or are even banned. However, there is a growing number of supporters of a solution to this controversy in which starch is recovered from the byproducts of its processing and, using such a source, it is modified using other bio-waste, both organic and inorganic. This approach is supported by raw material problems, the reduction in fossil resources and petroleum-based polymers, and problems related to the management of post-production wastes. One thing that is certain is that due to the multifunctional nature of starch and the dynamic development of methods for improving its properties, it will be a polymer of unflagging interest and application potential for many decades to come.

## Figures and Tables

**Table 1:** Characteristics of different types of starch.

Starch Type	Barley	Maize	Potato	Rice	Wheat	Tapioca
Source	Cereal	Cereal	Tuber	Cereal	Cereal	Root
Grain shape	Lenticular, round	Polyhedral, round	Round, oval	Polygonal, angular	Lenticular, round	Oval
Grain diameter [µm]	2–40	2–30	5–100	1–35	1–40	4–45
Polymorphism type	A	A	B	A	A	A/C
Amylose [%]	~22	23–32	18–29	~22	23–29	17–30
Amylopectin [%]	78	75	78	78	77	80
Lipid [%]	0.6–0.9	0.8	0.01	0.3	0.9	0.02
Protein [%]	0.1	0.35	0.1	0.3	0.4	0.1
Phosphorus [%]	0.05	0.09	0.20	0.09	0.3	0.009

**Table 2:** Examples of dual modification methods of starch based on hydrothermal treatments.

Type of Starch	Type of Dual Modification	Changes in Starch Properties	Reference
Corn	Infrared HMT	Decrease thermal stability, pasting properties, and viscosity	[46]
Wheat	Infrared HMT	Decrease thermal stability, pasting properties, and viscosity	[47]
Corn	Extrusion and HMT	Increase resistant starch content; decrease solubility and swelling power	[48]
Barley	Annealing and hydroxypropylation	Increase rheological properties, solubility, and freeze–thaw stability	[49]
Corn	Lactic, citric, and acetic acids and HMT	Increase resistant starch content, thermal stability, and rheological properties; decreased crystallinity	[50]
Corn	Lactic acid and HMT	Decrease viscosity; increase resistant content and thermal stability	[51]
Potato	Inclusion complexes with sodium stearate and HMT	Decrease swelling power and retrogradation; increase thermal stability and resistant fraction content	[52]

**Table 3:** Examples of preparation method of starch nanostructures.

Starch Nanostructure	Type of Starch	Method of Preparation	Min. Size [nm]	Reference
Nanocrystal	High-amylose maize	Acid-catalyzed hydrolysis	118	[58]
	Waxy	Acid-catalyzed hydrolysis	70	[59]
Nanofiber	High-amylose maize	Electrospinning	300	[60]
	High-amylose maize	Electrospinning	30	[61]
	Corn	Coaxial electrospinning	110	[62]
Nanogel	Potato	Graft copolymerization	120	[63]
	α-starch	Crosslinking	30	[64]
Nanomicelle	Starch octanoate	Esterification	410	[65]
	Waxy maize	Emulsification	60	[66]
	Corn	Graft copolymerization	20	[67]

**Table 4:** Development of new RS preparations over last decade.

Starch Source	Modification Conditions/Reagent	Type of RS	Reference
Unpeeled raw banana powder, peeled raw banana powder, and banana starch from Kluai Namwa Luang	Fruits were sliced, dried, and milled; starch was extracted from peeled raw banana powder	RS2	[90]
Lotus stem	Native starch digested with protease, lipase, α-amylase, and amyloglucosidase and subjected to ultrasonic treatment	RS2 + modification	[91]
Pea starch	Native; acid hydrolysis and pullulanase debranching	RS2 and RS3	[92]
Wrinkled and round pea starches	Heat–moisture treatment	RS3	[93]
Sago	Autoclaving, debranching by pullulanase, autoclaving, and cooling	RS3	[94]
Potato peels	Debranching by pullulanase, autoclaving, and cooling	RS3	[95]
Maize flour	Autoclaving in citrate buffer, protease, and amylase (+pullulanase in [96]) application, second autoclaving, and cooling	RS3	[96,97]
Pea and normal maize starches	Acid thinning, debranching, and recrystallization	RS3	[98]
Highly branched potato starch, waxy potato starch, amylomaltase-modified potato starch, and waxy rice starch	Debranching and recrystallization	RS3	[99,100]
*Cyperus esculentus* (tiger nut) starch	Debranching and nanoprecipitation	RS3	[101]
Sago starch	Autoclaving, debranching by pullulanase, autoclaving, and cooling (RS) and RS treated with 0.5 M hydrochloric acid	RS3	[102]
Cowpea starch	Autoclaving–cooling cycles (1, 3, and 5)	RS3	[103]
Waxy and normal maize starch	Gelatinized starches were subjected to temperature cycling between 4 and 30 °C (1 day at each temperature) or isothermal storage (4 °C) for 2 or 8 days	RS3	[104]
Highland barley, oat, and buckwheat starches	Enzymatic hydrolysis (α-amylase and pullulanase), autoclaving, and cooling	RS3	[105]
Waxy maize starch	Gelatinization, debranching by pullulanase, cooling, and self-assembly of nanoparticles	RS3	[106]
Potato starch	Gelatinization, debranching by pullulanase, autoclaving, and cooling	RS3	[107]
Different types of bean starches and maize starch	Autoclaving–cooling and α-amylase action or autoclaving and pullulanase	RS3	[108]
Sorghum starch and waxy rice starch	Heating/cooling with or without debranching gelatinized starch	RS3	[109]
High-amylose maize starches	Extrusion cooking with different moisture content	RS3	[110]
Culinary banana starch	Autoclaving or debranching with pullulanase	RS3	[111]
Waxy maize starch	Ultrasound-assisted annealing treatment of fractionated debranched starch	RS3	[112]
Fractionated lotus seed starch	Autoclaving–cooling and α-amylase and glucoamylase action	RS3	[113]
Cassava starch	Debranching by pullulanase and cooling	RS3	[114]
Maize flour and maize starch	Autoclaving–cooling treatments	RS3	[115]
Maize starch	Amylase and isoamylase hydrolysis and autoclaving–cooling	RS3	[116]
Lotus seed starch	Autoclaving–cooling or microwave heated/water bath heated, cooling, and purified by amylase and glucoamylase	RS3	[117,118]
Oat flour	Dual autoclaving–retrogradation treatment	RS3	[119]
Maize starch	1,4-maltotriohydrolase action, debranching using pullulanase, and autoclaving–cooling	RS3	[120]
Faba bean starch	Debranching using pullulanase and retrogradation treatment	RS3	[121]
Sweet potato, cassava, and high-amylose maize starches	Autoclaving in acetate buffer, debranching by amylase and pullulanase, autoclaving, and cooling	RS3	[122]
Maize and sorghum starches	Extrusion and different storage time	RS3	[123]
Pea starch	Ultrasonic treatment and cooling	RS3	[124]
Green banana flour	Autoclaving and debranching by pullulanase	RS3	[125]
Waxy proso millet grains	Debranching and retrogradation	RS3	[126]
Potato, wheat, corn, and tapioca starch	Retrogradation of starch and acetylation by acetic anhydride	RS3/RS4	[127,128]
Potato starch	Retrograded and acetylated starch produced via starch extrusion or starch hydrolysis with pullulanase	RS3/RS4	[129]
Potato starch	Retrograded and crosslinked by adipic acid	RS3/RS4	[130]
Potato starch	Retrograded, acetylated by acetic acid anhydride, and crosslinked by adipic acid	RS3/RS4	[131,132]
Cassava, potato, sweet potato, lentil, and banana	Octenyl succinic anhydride (OSA)	RS4	[133,134]
Canna	Acetic anhydride	RS4	[135]
Sorghum starch	Extrusion of phosphorylated starch	RS4	[136]
Pea starch	Crosslinking starch with sodium trimetaphosphate and sodium tripolyphosphate	RS4	[137]
Three Korean rice varieties	Crosslinking starch with sodium trimetaphosphate and sodium tripolyphosphate	RS4	[138]
Cassava and sweet potato roots, unripe banana, potato tubers, and lentil seeds	20, 40, and 60% of citric acid	RS4	[139]
Maize starch	Crosslinking starch with sodium trimetaphosphate and sodium tripolyphosphate under sonication and conventional conditions at various levels of pH	RS4	[140]
Potato starch	Extrusion combined with phosphorylation or succinylation	RS4	[141]
Waxy rice starch	Nanoparticles prepared by acid hydrolysis, crosslinking with sodium trimetaphosphate and freeze drying, freeze drying after sonication, and ethanol dehydration after sonication	RS4	[142]
High-amylose maize starch	Acetic anhydride, propionic anhydride, and butyric anhydride	RS4	[143,144]
Sweet potato starch	Microwave-assisted L-malic acid modification	RS4	[145]
Waxy rice starch	Lactic acid, phosphorylated, and dual-modified starch	RS4	[146]
Maize starch	Citric acid	RS4	[147]
Wheat flour	Acetic anhydride used for pulsed electric fields and conventional esterification	RS4	[148]
Cassava starch	Citric acid	RS4	[149]
Rice starch	Citric acid (10, 20, 30, 40%)	RS4	[150]
Waxy maize starch	Citrate esterification of debranched and non-debranched starch	RS4	[151]
Rice starch	Phosphorylation by using sodium trimetaphosphate and sodium tripolyphosphate	RS4	[152]
Oat starch	Acetic acid anhydride	RS4	[153]
Rice starch	Acetic acid anhydride	RS4	[154]
Field pea, faba bean, and maize starches	Phosphorus chloride and sodium trimetaphosphate/sodium tripolyphosphate in a semidry or aqueous state	RS4	[155]
Maize starch	L-malic acid	RS4	[156]
Rice starch	Pullulanase debranching and propionylation	RS4	[157]
Brown lentil starch	Addition of different lipids/fatty acids (10%, *w*/*w*) to both raw and cooked starch samples	RS5	[158]
White, black, and red rice	Rice was cooked with ghee, coconut oil, virgin coconut oil, and rice bran oil	RS5	[159]
Brown rice flour	Butyric, lauric, stearic, and linoleic acid complexation of amylose assisted by ultrasonication	RS5	[160]
Wheat starch	Lauric acid, stearic acid, and glycerol monolaurate; water bath or microwave oven	RS5	[161]
Arrowhead tubers	Starch and linoleic acid or stearic acid ultrasonic treatment	RS5	[162]
Yam starch	Palmitic acid	RS5	[163]
High-amylose maize starch	Debranching using pullulanase and complexation with stearic acid	RS5 + modification	[164]

**Table 5:** Progress in the dextrinization of starch over the last 10 years towards fiber and/or prebiotic preparations.

Type of Dextrin/Maltodextrin	Heating Conditions	Acid Catalyst	Ref.
Banana maltodextrin	90–110 °C, 1–3 h	2.2 M HCl to 80:1, 120:1, or 160:1 (*w*/*v*) starch–acid proportion	[175]
Banana maltodextrin	90 °C, 1 h	2.2 M HCl to 160:1 (*w*/*v*) starch–acid proportion	[176]
Barley dextrin	90 °C, 1 h	2.2 M HCl	[177]
Breadfruit dextrin	140 °C, 3 h	2.2 M HCl or 1.32 M CH_3_COOH to 1.82 g of acid/kg db starch ratio	[178]
Cassava dextrin	100–120 °C, 1–3 h	0.04–0.1% HCl	[179]
Cassava dextrin and cassava maltodextrin	120 °C, 1–3 h	0.04–0.06% HCl	[166]
Cassava dextrin and Maltodextrin	90–110 °C, 1–3 h	2.2 M HCl to 80:1, 120:1, or 160:1 (*w*/*v*) starch–acid proportion	[180]
Cassava dextrin and Maltodextrin	90 °C, 3 h	2.2 M HCl to 160:1 (*w*/*v*) starch–acid proportion	[171]
Maize dextrin	140–200 °C, 2 h	0.5 M HCl to pH 3.0	[167]
Maize dextrin	120–140 °C or 140–180 °C, 3 h	0.05–0.2% of HCl or 0.5–2.5% of acetic acid	[181]
Maize dextrin	180 °C, 0.5, 3 or 5 h	0.5 M HCl to pH 3.0	[182]
Maize dextrin	180 °C, 1–4 h	0.5 M HCl to pH 3.0	[183]
Maize maltodextrin	140–160 °C, 1.5–2 h	1 mL of 0.5% citric acid/20 g starch	[184]
Normal and waxy tapioca dextrin	130–170 °C, 1–4 h	0.5 M HCl to pH 3.0	[168,185]
Potato dextrin	40–120 W, 1 or 10 cycles, 15–90 s	0.1% of HCl + 0.1% of citric acid	[170]
Potato dextrin	130 °C, 4 h	0.1% of HCl + 0.1% of citric acid	[186]
Potato dextrin	735–1050 W, 2–10 min	0.1% of HCl + 0.1% of citric acid	[169]
Potato dextrin	105–630W, 2–10 min	0.1% of HCl + 0.1% of citric acid	[187]
Potato dextrin	150 °C, 3 h or 180 °C, 1 h	0.1% of HCl + 0.1% of citric acid	[188]
Potato dextrin	130 °C, 2 h	0.1% of HCl + 40% of tartaric acid	[189,190]
Potato dextrin	130 °C, 3 h or 130 °C, 2 h	0.1% of HCl + 0.1% of citric acid or 0.1% of HCl + 40% of tartaric acid	[191]
Rice dextrin	170 °C for 350 min	Without an acidic catalyst	[192]
Sorghum dextrin	120 °C, 6 h	0.182% HCl	[193]
Waxy maize dextrin	150–170 °C, 1–10 h	0.036–0.144% HCl	[165]
Waxy maize dextrin	170 °C, 0.5–4 h	0.5 M HCl to pH 3.0	[194,195,196]
Waxy maize dextrin	170 °C, 4 h	0.5 M HCl to pH 3.0	[174]
Waxy maize dextrin	150 or 170 °C, 0.5–4 h	0.5 M HCl to pH 3.0 or pH 2.0	[197]
Waxy maize dextrin	150 or 170 °C, 4 h	0.5 M HCl to pH 3.0 or pH 2.0	[198]
Yam dextrin	140 °C, mostly 1.5–4.5 h	Mostly 0.99–2.65 g HCl/g starch db	[199]

## Data Availability

Not applicable.

## References

[1] Das A., Ringu T., Ghosh S., Pramanik N. (2023). A comprehensive review on recent advances in preparation, physicochemical characterization, and bioengineering applications of biopolymers. Polym. Bull..

[2] Baranwal J., Barse B., Fais A., Delogu G.L., Kumar A. (2022). Biopolymer: A Sustainable Material for Food and Medical Applications. Polymers.

[3] Globe Newswire. https://www.globenewswire.com/en/news-release/2023/02/28/2617539/28124/en/Starch-Global-Market-to-Reach-199-8-Million-Metric-Tons-by-2030-Use-of-Starch-as-a-Fat-Replacer-Drives-Growth.html.

[4] Maximize Market Research Pvt. Ltd. https://www.maximizemarketresearch.com/market-report/global-starch-market/112948/.

[5] Tegge G. (2010). Skrobia i Jej Pochodne.

[6] Pérez S., Bertoft E. (2010). The molecular structures of starch components and their contribution to the architecture of starch granules: A comprehensive review. Starch Stärke.

[7] Vamadevan V., Bertoft E. (2014). Structure-function relationships of starch components. Starch Stärke.

[8] Bertoft E., Piyachomkwan K., Chatakanonda P., Sriroth K. (2008). Internal unit chain composition in amylopectins. Carbohydr. Polym..

[9] Robyt J.F. (2008). Starch: Structure, Properties, Chemistry and Enzymology. Glycoscience.

[10] Alcázar-Alay S.C., Meireles M.A.A. (2015). Physicochemical properties, modifications and applications of starches from different botanical sources. Food Sci. Technol..

[11] Hoover R. (2001). Composition, molecular structure, and physicochemical properties of tuber and root starches: A review. Carbohydr. Polym..

[12] Yusuph M., Tester R.F., Ansell R., Snape C.E. (2003). Composition and properties of starches extracted from tubers of different potato varieties grown under the same environmental conditions. Food Chem..

[13] Srichuwong S., Sunarti T.C., Mishima T., Isono N., Hisamatsu M. (2005). Starches from different botanical sources I: Contribution of amylopectin fine structure to thermal properties and enzyme digestibility. Carbohydr. Polym..

[14] Mitrus M., Wojtowicz A., Moscicki L. (2009). Biodegradable Polymers and Their Practical Utility. Thermoplastic Starch: A Green Material for Various Industries.

[15] Matsushima R. (2015). Morphological Variations of Starch Grains. Starch.

[16] Bertoft E. (2017). Understanding Starch Structure: Recent Progress. Agronomy.

[17] Kong L., Lee C., Kim S., Ziegler G. (2014). Characterization of Starch Polymorphic Structures Using Vibrational Sum Frequency Generation Spectroscopy. J. Phys. Chem. B.

[18] Bashir K., Aggarwal M. (2019). Physicochemical, structural and functional properties of native and irradiated starch: A review. J. Food. Sci. Technol..

[19] Ai Y., Jane J. (2018). Understanding Starch Structure and Functionality. Woodhead Publishing Series in Food Science, Technology and Nutrition, Starch in Food.

[20] Carlstedt J., Wojtasz J., Fyhr P., Kocherbitov V. (2015). Understanding starch gelatinization: The phase diagram approach. Carbohyd. Polym..

[21] Ai Y., Jane J. (2015). Gelatinization and rheological properties of starch. Starch Starke.

[22] Singh S., Singh N., Isono N., Noda T. (2010). Relationship of granule size distribution and amylopectin structure with pasting, thermal, and retrogradation properties in wheat starch. J. Agric. Food Chem..

[23] Singh N., Singh J., Kaur L., Sodhi N.S., Gill B.S. (2003). Morphological, thermal and rheological properties of starches from different botanical sources. Food Chem..

[24] Gao J., Luo Z.G., Luo F.X. (2012). Ionic liquids as solvents for dissolution of corn starch and homogeneous synthesis of fatty-acid starch esters without catalysts. Carbohyd. Polym..

[25] Fan Y., Picchioni F. (2020). Modification of starch: A review on the application of “green” solvents and controlled functionalization. Carbohyd. Polym..

[26] El Seoud O.A., Koschella A., Fidale L.C., Dorn S., Heinze T. (2007). Applications of ionic liquids in carbohydrate chemistry: A window of opportunities. Biomacromolecules.

[27] Ptak S., Zarski A., Kapusniak J. (2020). The Importance of Ionic Liquids in the Modification of Starch and Processing of Starch-Based Materials. Materials.

[28] Wilpiszewska K., Spychaj T. (2011). Ionic liquids: Media for starch dissolution, plasticization and modification. Carbohyd. Polym..

[29] Pawłowska B., Telesiński A., Biczak R. (2019). Phytotoxicity of ionic liquids. Chemosphere.

[30] Gonçalves A.R.P., Paredes X., Cristino A.F., Santos F.J.V., Queirós C.S.G.P. (2021). Ionic Liquids-A Review of Their Toxicity to Living Organisms. Int. J. Mol. Sci..

[31] Kaar J.L., Jesionowski A.M., Berberich J.A., Moulton R., Russell A.J. (2003). Impact of ionic liquid physical properties on lipase activity and stability. J. Am. Chem. Soc..

[32] Muljana H., Picchioni F., Heeres H.J., Janssen L.P.B.M. (2009). Supercritical carbon dioxide (scCO_2_) induced gelatinization of potato starch. Carbohyd. Polym..

[33] Zaidul I.S.M., Noda T., Sharif K.M., Karim A.A., Smith R.L. (2014). Reduction of gelatinization temperatures of starch blend suspensions with supercritical CO_2_ treatment. J. Supercrit. Fluids.

[34] Muljana H., van der Knoop S., Keijzer D., Picchioni F., Janssen L.P.B.M., Heeres H.J. (2010). Synthesis of fatty acid starch esters in supercritical carbon dioxide. Carbohyd. Polym..

[35] Muljana H., Picchioni F., Knez Z., Heeres H.J., Janssen L.P.B.M. (2011). Insights in starch acetylation in sub- and supercritical CO_2_. Carbohyd. Res..

[36] Salimi K., Yilmaz M., Rzayev Z.M., Piskin E. (2014). Controlled graft copolymerization of lactic acid onto starch in a supercritical carbon dioxide medium. Carbohyd. Polym..

[37] Ayoub A., Rizvi S.S.H. (2008). Properties of supercritical fluid extrusion-based crosslinked starch extrudates. J. Appl. Polym. Sci..

[38] Ayoub A., Rizvi S.S.H. (2011). Reactive supercritical fluid extrusion for development of moisture resistant starch-based foams. J. Appl. Polym. Sci..

[39] Manoi K., Rizvi S.S.H. (2010). Physicochemical characteristics of phosphorylated cross-linked starch produced by reactive supercritical fluid extrusion. Carbohyd. Polym..

[40] Flieger J., Flieger M. (2020). Ionic Liquids Toxicity-Benefits and Threats. Int. J. Mol. Sci..

[41] Hu X.-P., Zhang B., Jin Z.-Y., Xu X.-M., Chen H.-Q. (2017). Effect of high hydrostatic pressure and retrogradation treatments on structural and physicochemical properties of waxy wheat starch. Food Chem..

[42] La Fuente C.I., de Souza A.T., Tadini C.C., Augusto P.E.D. (2019). Ozonation of cassava starch to produce biodegradable films. Int. J. Biol. Macromol..

[43] Sifuentes-Nieves I., Neira-Velázquez G., Hernández-Hernández E., Barriga-Castro E., Gallardo-Vega C., Velazquez G., Mendez-Montealvo G. (2019). Influence of gelatinization process and HMDSO plasma treatment on the chemical changes and water vapor permeability of corn starch films. Int. J. Biol. Macromol..

[44] Sifuentes-Nieves I., Velazquez G., Flores-Silva P.C., Hernández-Hernández E., Neira-Velázquez G., Gallardo-Vega C., Mendez- Montealvo G. (2020). HMDSO plasma treatment as alternative to modify structural properties of granular starch. Int. J. Biol. Macromol..

[45] Wu Z., Qiao D., Zhao S., Lin Q., Zhang B., Xie F. (2022). Nonthermal physical modification of starch: An overview of recent research into structure and property alterations. Int. J. Biol. Macromol..

[46] Ismailoglu S.O., Basman A. (2015). Effects of infrared heat-moisture treatment on physicochemical properties of corn starch. Starch Starke.

[47] Ismailoglu S.O., Basman A. (2016). Physicochemical properties of infrared heat moisture treated wheat starch. Starch-Starke.

[48] Yan X., Wu Z.Z., Li M.Y., Yin F., Ren K.X., Tao H. (2019). The combined effects of extrusion and heat-moisture treatment on the physicochemical properties and digestibility of corn starch. Int. J. Biol. Macromol..

[49] Devi R., Sit N. (2019). Effect of single and dual steps annealing in combination with hydroxypropylation on physicochemical, functional and rheological properties of barley starch. Int. J. Biol. Macromol..

[50] Maior L.O., Almeida V.S., Barretti B.R.V., Ito V.C., Beninca C., Demiate I.M., Lacerda L.G. (2020). Combination of organic acid and heat–moisture treatment: Impact on the thermal, structural, pasting properties and digestibility of maize starch. J. Therm. Anal. Calorim..

[51] Reyes I., Hernandez-Jaimes C., Meraz M., Vernon-Carter E.J., Alvarez-Ramirez J. (2021). Effect of combined heat-moisture/lactic acid treatment on the physicochemical and in vitro digestibility properties of corn starch. Starch Starke.

[52] Yassaroh Y., Nurhaini F.F., Woortman A.J.J., Loos K. (2021). Physicochemical properties of heat-moisture treated, sodium stearate complexed starch: The effect of sodium stearate concentration. Carbohyd. Polym..

[53] Fonseca L.M., El Halal S.L.M., Dias A.R.G., da Rosa Zavareze E. (2021). Physical modification of starch by heat-moisture treatment and annealing and their applications: A review. Carbohyd. Polym..

[54] García-Guzmán L., Cabrera-Barjas G., Soria-Hernández C.G., Castaño J., Guadarrama-Lezama A.Y., Rodríguez Llamazares S. (2022). Progress in Starch-Based Materials for Food Packaging Applications. Polysaccharides.

[55] Ghosh S., Sinha J.K., Ghosh S., Vashisth K., Han S., Bhaskar R. (2023). Microplastics as an Emerging Threat to the Global Environment and Human Health. Sustainability.

[56] Zarski A., Bajer K., Kapuśniak J. (2021). Review of the Most Important Methods of Improving the Processing Properties of Starch toward Non-Food Applications. Polymers.

[57] Muñoz-Gimena P.F., Oliver-Cuenca V., Peponi L., López D. (2023). A Review on Reinforcements and Additives in Starch-Based Composites for Food Packaging. Polymers.

[58] Le Corre D., Bras J., Dufresne A. (2010). Starch nanoparticles: A review. Biomacromolecules.

[59] Dufresne A., Castaño J. (2017). Polysaccharide nanomaterial reinforced starch nanocomposites: A review. Starch Stärke.

[60] Wang W., Jin X., Zhu Y., Zhu C., Yang J., Wang H., Lin T. (2016). Effect of vapor-phase glutaraldehyde crosslinking on electrospun starch fibers. Carbohydr. Polym..

[61] Wu D., Samanta A., Srivastava R.K., Hakkarainen M. (2017). Starch-Derived Nanographene Oxide Paves the Way for Electrospinnable and Bioactive Starch Scaffolds for Bone Tissue Engineering. Biomacromolecules.

[62] Komur B., Bayrak F., Ekren N., Eroglu M., Oktar F.N., Sinirlioglu Z., Yucel S., Guler O., Gunduz O. (2017). Starch/PCL composite nanofibers by coaxial electrospinning technique for biomedical applications. Biomed. Eng. Online.

[63] Noh G.J., Lim S.A., Lee E.S. (2019). pH-responsive squeezing polysaccharidic nanogels for efficient docetaxel delivery. Polym. Adv. Technol..

[64] Liang T., Hou J., Qu M., Zhao M., Raj I. (2020). High-viscosity α—Starch nanogel particles to enhance oil recovery. RSC Adv..

[65] Ying X., Shan C., Jiang K., Chen Z., Du Y. (2014). Intracellular pH-sensitive delivery CaCO_3_ nanoparticles templated by hydrophobic modified starch micelles. RSC Adv..

[66] Liu W., Li Y., Goff H.D., Nsor-Atindana J., Ma J., Zhong F. (2019). Interfacial activity and self-assembly behavior of dissolved and granular octenyl succinate anhydride starches. Langmuir.

[67] Wang X., Liu Z., Huang L. (2019). pH and thermo dual-responsive starch-g-P(DEAEMA-co-PEGMA): Synthesis via SET-LRP, selfassembly and drug release behaviors. React. Funct. Polym..

[68] Kumari A., Yadav S.K., Yadav S.C. (2010). Biodegradable polymeric nanoparticles based drug delivery systems. Colloids Surf. B Biointerfaces.

[69] Chen Y.F., Kaur L., Singh J., Sjoo M., Nilsson L. (2018). Chemical Modification of Starch. Starch in Food.

[70] Amaraweera S.M., Gunathilake C., Gunawardene O.H.P., Fernando N.M.L., Wanninayaka D.B., Dassanayake R.S., Rajapaksha S.M., Manamperi A., Fernando C.A.N., Kulatunga A.K. (2021). Development of Starch-Based Materials Using Current Modification Techniques and Their Applications: A Review. Molecules.

[71] Shokri Z., Seidi F., Reza Saeb M., Jin Y., Li C., Xiao H. (2022). Elucidating the impact of enzymatic modifications on the structure, properties, and applications of cellulose, chitosan, starch and their derivatives: A review. Mater. Today Chem..

[72] Tomasik P., Schilling C.H. (2004). Chemical modification of starch. Adv. Carbohydr. Chem. Biochem..

[73] Wang L., Qu L., Wu Y., Men Y., Liu Z. (2017). Synthesis of regioselective starch based macroinitiators at molecular level. Starch Starke.

[74] Tan W., Li Q., Gao Z., Qiu S., Dong F., Guo Z. (2017). Design, synthesis of novel starch derivative bearing 1,2,3-triazolium and pyridinium and evaluation of its antifungal activity. Carbohyd. Polym..

[75] Brownlee I.A., Gill S., Wilcox M.D., Pearson J.P., Chater P.I. (2018). Starch digestion in the upper gastrointestinal tract of humans. Starke.

[76] Birt D.F., Boylston T., Hendrich S., Jane J.L., Hollis J., Li L., McClelland J., Moore S., Phillips G.J., Rowling M. (2013). Resistant starch: Promise for improving human health. Adv. Nutr..

[77] Cerqueira F.M., Photenhauer A.L., Pollet R.M., Brown H.A. (2019). Starch Digestion by Gut Bacteria: Crowdsourcing for Carbs. Trends Microbiol..

[78] Fuller S., Beck E., Salman H., Tapsell L. (2016). New Horizons for the Study of Dietary Fiber and Health: A Review. Plant Foods Hum. Nutr..

[79] Dhingra D., Michael M., Rajput H., Patil R.T. (2012). Dietary fibre in foods: A review. J. Food Sci. Technol..

[80] Granato D., Barba F.J., Kovačević D.B., Lorenzo J.M., Cruz A.G., Putnik P. (2020). Functional Foods: Product Development, Technological Trends, Efficacy Testing, and Safety. Annu. Rev. Food Sci. Technol..

[81] Gibson G., Hutkins R., Sanders M.E., Prescott S.L., Reimer R.A., Salminen S.J., Scott K., Stanton C., Swanson K.S., Cani P.D. (2017). Expert consensus document: The International Scientific Association for Probiotics and Prebiotics (ISAPP) consensus statement on the definition and scope of prebiotics. Nat. Rev. Gastroenterol. Hepatol..

[82] Kraithong S., Wang S., Junejo S.A., Fu X., Theppawong A., Zhang B., Huang Q. (2022). Type 1 resistant starch: Nutritional properties and industry applications. Food Hydrocoll..

[83] Bendiks Z.A., Knudsen K.E.B., Keenan M.J., Marco M.L. (2020). Conserved and variable responses of the gut microbiome to resistant starch type 2. Nutr. Res..

[84] Lockyer S., Nugent A.P. (2017). Health effects of resistant starch. Nutr. Bull..

[85] Raigond P., Ezekiel R., Raigond B. (2015). Resistant starch in food: A review. J. Sci. Food Agric..

[86] Gutiérrez T.J., Tovar J. (2021). Update of the concept of type 5 resistant starch (RS5): Self-assembled starch V-type complexes. Trends Food Sci. Technol..

[87] Ze X., Duncan S.H., Louis P., Flint H.J. (2012). *Ruminococcus bromii* is a keystone species for the degradation of resistant starch in the human colon. ISME J..

[88] Deehan E.C., Yang C., Perez-Muñoz M.E., Nguyen N.K., Cheng C.C., Triador L., Zhang Z., Bakal J.A., Walter J. (2020). Precision Microbiome Modulation with Discrete Dietary Fiber Structures Directs Short-Chain Fatty Acid Production. Cell Host Microbe.

[89] Do M.H., Seo Y.S., Park H.-Y. (2020). Polysaccharides: Bowel health and gut microbiota. Crit. Rev. Food Sci..

[90] Jaiturong P., Laosirisathian N., Sirithunyalug B., Eitssayeam C., Sirilun S., Chaiyana W., Sirithunyalug J. (2020). Physicochemical and prebiotic properties of resistant starch from *Musa sapientum* Linn., ABB group, cv. Kluai Namwa Luang. Heliyon.

[91] Noor N., Gani A., Jhan F., Jenno J.L.H., Dar M.A. (2021). Resistant starch type 2 from lotus stem: Ultrasonic effect on physical and nutraceutical properties. Ultrason. Sonochem..

[92] Chang D., Hu X., Ma Z. (2022). Pea-Resistant Starch with Different Multi-scale Structural Features Attenuates the Obesity-Related Physiological Changes in High-Fat Diet Mice. J. Agric. Food Chem..

[93] Cheng F., Ren Y., Warkentin T.D., Ai Y. (2024). Heat-Moisture Treatment to Modify Structure and Functionality and Reduce Digestibility of Wrinkled and Round Pea Starches. Carbohydr. Polym..

[94] Rashid R.S.A., Mohamed A.M.D., Achudan S.N., Mittis P. (2020). Physicochemical properties of resistant starch type III from sago starch at different palm stages. Mater. Today Proc..

[95] Thakur M., Rai A.K., Mishra B.B., Singh S.P. (2021). Novel insight into valorization of potato peel biomass into type III resistant starch and maltooligosaccharide molecules. Environ. Technol. Innov..

[96] Khan A., Siddiqui S., Rahman U.U., Ali H., Saba M., Azhar F.A., Rehman M.M.U., Shah A.A., Badshah M., Hasan F. (2020). Physicochemical properties of enzymatically prepared resistant starch from maize flour and its use in cookies formulation. Int. J. Food Prop..

[97] Khan A., Rahman U.U., Siddiqui S., Irfan M., Shah A.A., Badshah M., Hasan F., Khan S. (2019). Preparation and characterization of resistant starch type III from enzymatically hydrolyzed maize flour. Mol. Biol. Rep..

[98] Li L., Yuan T.Z., Ai Y. (2020). Development, structure and in vitro digestibility of type 3 resistant starch from acid-thinned and debranched pea and normal maize starches. Food Chem..

[99] Klostermann C.E., Buwalda P.L., Leemhuis H., de Vos P., Schols H.A., Bitter J.H. (2021). Digestibility of resistant starch type 3 is affected by crystal type, molecular weight and molecular weight distribution. Carbohyd. Polym..

[100] Silva Lagos L., Klostermann C.E., López-Velázquez G., Fernández-Lainez C., Leemhuis H., Oudhuis A.A.C.M.L., Buwalda P., Schols H.A., de Vos P. (2024). Crystal Type, Chain Length and Polydispersity Impact the Resistant Starch Type 3 Immunomodulatory Capacity via Toll-like Receptors. Carbohyd. Polym..

[101] Yan X., Diao M., Yu Y., Gao F., Wang E., Wang Z., Zhang T., Zhao P. (2022). Characterization of resistant starch nanoparticles prepared via debranching and nanoprecipitation. Food Chem..

[102] Zi-Ni T., Rosma A., Napisah H., Karim A.A., Liong M.-T. (2015). Characteristics of *Metroxylon sagu* Resistant Starch Type III as Prebiotic Substance. J. Food Sci..

[103] Ratnaningsih N., Suparmo, Harmayani E., Marsono Y. (2020). Physicochemical properties, in vitro starch digestibility, and estimated glycemic index of resistant starch from cowpea (*Vigna unguiculata*) starch by autoclaving-cooling cycles. Int. J. Biol. Macromol..

[104] Zhou X., Chung H.-J., Kim J.-Y., Lim S.-T. (2013). In vitro analyses of resistant starch in retrograded waxy and normal corn starches. Int. J. Biol. Macromol..

[105] Shen R.-L., Zhang W.-J., Dong J.-L. (2016). Preparation, structural characteristics and digestibility of resistant starches from highland barley, oats and buckwheat starches. J. Food Nutr. Res..

[106] Adra H.J., Zhi J., Luo K., Kim Y.R. (2022). Facile Preparation of Highly Uniform Type 3 Resistant Starch Nanoparticles. Carbohydr. Polym..

[107] Thakur M., Sharma N., Rai A.K., Singh S.P. (2021). A novel cold-active type I pullulanase from a hot-spring metagenome for effective debranching and production of resistant starch. Bioresour. Technol..

[108] Simons C.W., Hall C., Vatansever S. (2018). Production of resistant starch (RS3) from edible bean starches. J. Food Process Preserv..

[109] Giuberti G., Gallo A. (2020). In vitro evaluation of fermentation characteristics of type 3 resistant starch. Heliyon.

[110] Masatcioglu T.M., Sumer Z., Koksel H. (2017). An innovative approach for significantly increasing enzyme resistant starch type 3 content in high amylose starches by using extrusion cooking. J. Cereal Sci..

[111] Khawas P., Deka S.C. (2017). Effect of modified resistant starch of culinary banana on physicochemical, functional, morphological, diffraction, and thermal properties. Int. J. Food Prop..

[112] Chang R., Lu H., Bian X., Tian Y., Jin Z. (2021). Ultrasound assisted annealing production of resistant starches type 3 from fractionated debranched starch: Structural characterization and in-vitro digestibility. Food Hydrocoll..

[113] Zeng H., Chen P., Chen C., Huang C., Lin S., Zheng B., Zhang Y. (2018). Structural properties and prebiotic activities of fractionated lotus seed resistant starches. Food Chem..

[114] Sari P.M., Uttapap D., Wandee Y., Kotatha D., Udchumpisai W., Puttanlek C., Rungsardthong V. (2022). Powder structure and gelation behaviour of debranched cassava starches prepared with and without incubation. Int. J. Food Sci. Technol..

[115] Faridah D.N., Frista S.R., Dias I., Ahmad A.F., Anuraga J., Putri A.M. (2022). Verification of autoclaving-cooling treatment to increase the resistant starch contents in food starches based on meta-analysis result. Front. Nutr..

[116] Li Y., Xu J., Zhang L., Ding Z., Gu Z., Shi G. (2017). Investigation of debranching pattern of a thermostable isoamylase and its application for the production of resistant starch. Carbohyd Res..

[117] Zhang Y., Wang Y., Zheng B., Lu X., Zhuang W. (2013). The in vitro effects of retrograded starch (resistant starch type 3) from lotus seed starch on the proliferation of *Bifidobacterium adolescentis*. Food Funct..

[118] Huang Y., Yang S., Huang Z., Yuan Y., Miao S., Zhang Y., Zeng H., Zheng B., Deng K. (2022). Difference in the adhesion of *Bifidobacterium breve* to lotus seed resistant starch is attributable to its structural performance conferred by the preparation method. Int. J. Biol. Macromol..

[119] Shah A., Masoodi F.A., Gani A., Ashwar B.A. (2016). In-vitro digestibility, rheology, structure, and functionality of RS3 from oat starch. Food Chem..

[120] Wu C., Zhou X., Wei B., Tian Y., Xu X., Jin Z. (2017). Effects of *α*-maltotriohydrolase hydrolysis prior to debranching on the structure and digestibility of normal maize starch. Starch Stärke.

[121] Suárez-Diéguez T., Pérez-Moreno F., Ariza-Ortega J.A., López-Rodríguez G., Nieto J.A. (2021). Obtention and characterization of resistant starch from creole faba bean (*Vicia faba* L. creole) as a promising functional ingredient. LWT.

[122] Villas-Boas F., Facchinatto W.M., Colnago L.A., Volanti D.P., Franco C.M.L. (2020). Effect of amylolysis on the formation, the molecular, crystalline and thermal characteristics and the digestibility of retrograded starches. Int. J. Biol. Macromol..

[123] Shaikh F., Ali T.M., Mustafa G., Hasnain A. (2020). Structural, functional and digestibility characteristics of sorghum and corn starch extrudates (RS3) as affected by cold storage time. Int. J. Biol. Macromol..

[124] You Q., Zhang X., Fang X., Yin X., Luo C., Wan M. (2019). Ultrasonic-Assisted Preparation and Characterization of RS3 from Pea Starch. Food Bioprocess. Technol..

[125] Liao H.-J., Hung C.-C. (2015). Chemical composition and in vitro starch digestibility of green banana (cv. Giant Cavendish) flour and its derived autoclaved/debranched powder. LWT Food Sci. Technol..

[126] Gong M., Li X., Xiong L., Sun Q. (2016). Retrogradation property of starch nanoparticles prepared by pullulanase and recrystallization. Starch Stärke.

[127] Zięba T., Kapelko M., Szumny A. (2013). Effect of preparation method on the properties of potato starch acetates with an equal degree of substitution. Carbohyd. Polym..

[128] Zięba T., Kapelko-Żeberska M., Gryszkin A., Wilczak A., Raszewski B., Spychaj R. (2019). Effect of the Botanical Origin on Properties of RS3/4 Type Resistant Starch. Polymers.

[129] Kapelko M., Zięba T., Gryszkin A., Styczyńska M., Wilczak A. (2013). Properties of retrograded and acetylated starch produced via starch extrusion or starch hydrolysis with pullulanase. Carbohydr. Polym..

[130] Kapelko M., Zięba T., Michalski A., Gryszkin A. (2015). Effect of cross-linking degree on selected properties of retrograded starch adipate. Food Chem..

[131] Kapelko-Żeberska M., Zięba T., Spychaj R., Gryszkin A. (2015). Acetylated adipate of retrograded starch as RS 3/4 type resistant starch. Food Chem..

[132] Kapelko-Żeberska M., Zięba T., Spychaj R., Gryszkin A. (2017). Selected Rheological Properties of RS3/4 Type Resistant Starch. Pol. J. Food Nutr. Sci..

[133] Remya R., Jyothi A.N., Sreekumar J. (2017). Comparative study of RS4 type resistant starches derived from cassava and potato starches via octenyl succinylation. Starch Stärke.

[134] Remya R., Jyothi A.N., Sreekumar J. (2018). Morphological, structural and digestibility properties of RS4 enriched octenyl succinylated sweet potato, banana and lentil starches. Food Hydrocoll..

[135] Carolina A., Ilmi F. (2016). Production of Indonesian Canna edulis Type IV resistant starch through acetylation modification. Int. Food Res. J..

[136] Escobar-Puentes A., Rincón S., García-Gurrola A., Zepeda A., Calvo-López A.D., Martínez-Bustos F. (2019). Development of a Third-Generation Snack with Type 4 Resistant Sorghum Starch: Physicochemical and Sensorial Properties. Food Biosci..

[137] Shi M., Gu F., Wu J., Yu S., Gao Q. (2013). Preparation, physicochemical properties, and in vitro digestibility of cross-linked resistant starch from pea starch. Starch Stärke.

[138] Yu M., Shin M. (2015). Improving gel formation of rice starch added with cross-linked resistant starch prepared from rice starch. Starch Stärke.

[139] Remya R., Jyothi A.N., Sreekumar J. (2018). Effect of Chemical Modification with Citric Acid on the Physicochemical Properties and Resistant Starch Formation in Different Starches. Carbohydr. Polym..

[140] Falsafi S.R., Maghsoudlou Y., Aalami M., Jafari S.M., Raeisi M. (2019). Physicochemical and Morphological Properties of Resistant Starch Type 4 Prepared under Ultrasound and Conventional Conditions and Their In-Vitro and In-Vivo Digestibilities. Ultrason. Sonochem..

[141] Calvo-López A.D., Martínez-Bustos F. (2017). Optimization of Extrusion Process of Directly Expanded Snacks Based on Potato Starch in a Single Step for the Formation of Type IV Resistant Starch. Plant Foods Hum. Nutr..

[142] Jeong O., Shin M. (2018). Preparation and Stability of Resistant Starch Nanoparticles, Using Acid Hydrolysis and Cross-Linking of Waxy Rice Starch. Food Chem..

[143] Li M., Wang F., Wang J., Wang R., Strappe P., Zheng B., Zhou Z., Chen L. (2021). Manipulation of the Internal Structure of Starch by Propionyl Treatment and Its Diverse Influence on Digestion and in Vitro Fermentation Characteristics. Carbohydr. Polym..

[144] Li M., Wang F., Wang J., Wang A., Yao X., Strappe P., Zhou Z., Wu Q., Guo T. (2022). Starch Acylation of Different Short-Chain Fatty Acids and Its Corresponding Influence on Gut Microbiome and Diabetic Indexes. Food Chem..

[145] Wang W., Hu A., Li J., Liu G., Wang M., Zheng J. (2022). Comparison of Physicochemical Properties and Digestibility of Sweet Potato Starch after Two Modifications of Microwave Alone and Microwave-Assisted L-Malic Acid. Int. J. Biol. Macromol..

[146] Cao C., Wei D., Xuan F., Deng C., Hu J., Zhou Y. (2022). Comparative Study on the Structure and Physicochemical of Waxy Rice Starch by Phosphorylation, Lactylation and Dual-Modified. Food Sci. Technol..

[147] Hedayati S., Niakousari M. (2018). Microstructure, Pasting and Textural Properties of Wheat Starch-Corn Starch Citrate Composites. Food Hydrocoll..

[148] Hong J., An D., Zeng X.A., Han Z., Zheng X., Cai M., Bian K., Aadil R.M. (2020). Behaviors of Large A-Type and Small B-Type Wheat Starch Granules Esterified by Conventional and Pulsed Electric Fields Assisted Methods. Int. J. Biol. Macromol..

[149] Oltramari K., Madrona G.S., Neto A.M., de Morais G.R., Baesso M.L., Bergamasco R., de Moraes F.F. (2017). Citrate esterified cassava starch: Preparation, physicochemical characterisation, and application in dairy beverages. Starch Stärke.

[150] Zhong C., Xiong Y., Lu H., Luo S., Wu J., Ye J., Liu C. (2022). Preparation and Characterization of Rice Starch Citrates by Superheated Steam: A New Strategy of Producing Resistant Starch. LWT.

[151] Liu Y., Liu J., Kong J., Wang R., Liu M., Strappe P., Blanchard C., Zhou Z. (2020). Citrate esterification of debranched waxy maize starch: Structural, physicochemical and amylolysis properties. Food Hydrocoll..

[152] Ashwar B.A., Gani A., Shah A., Masoodi F.A. (2017). Physicochemical Properties, in-Vitro Digestibility and Structural Elucidation of RS4 from Rice Starch. Int. J. Biol. Macromol..

[153] Shah A., Masoodi F.A., Gani A., Ashwar B.A. (2017). Physicochemical, Rheological and Structural Characterization of Acetylated Oat Starches. LWT.

[154] Ashwar B.A., Gani A., Shah A., Masoodi F.A. (2017). Production of RS4 from rice by acetylation: Physico-chemical, thermal, and structural characterization. Starch Stärke.

[155] Dong H., Vasanthan T. (2020). Amylase Resistance of Corn, Faba Bean, and Field Pea Starches as Influenced by Three Different Phosphorylation (Cross-Linking) Techniques. Food Hydrocoll..

[156] Tian S.-Q., Wang Z.-L., Wang X.-W., Zhao R.-Y. (2016). Development and digestion of resistant malate starch produced by L-malic acid treatment. RSC Adv..

[157] Wang R., Li M., Liu J., Wang F., Wang J., Zhou Z. (2021). Dual Modification Manipulates Rice Starch Characteristics Following Debranching and Propionate Esterification. Food Hydrocoll..

[158] Okumus B.N., Tacer-Caba Z., Kahraman K., Nilufer-Erdil D. (2018). Resistant Starch Type V Formation in Brown Lentil (Lens Culinaris Medikus) Starch with Different Lipids/Fatty Acids. Food Chem..

[159] Krishnan V., Mondal D., Bollinedi H., Srivastava S., SV R., Madhavan L., Thomas B., R A.T., Singh A., Singh A.K. (2020). Cooking Fat Types Alter the Inherent Glycaemic Response of Niche Rice Varieties through Resistant Starch (RS) Formation. Int. J. Biol. Macromol..

[160] Chumsri P., Panpipat W., Cheong L.-Z., Chaijan M. (2022). Formation of Intermediate Amylose Rice Starch–Lipid Complex Assisted by Ultrasonication. Foods.

[161] Kang X., Jia S., Gao W., Wang B., Zhang X., Tian Y., Sun Q., Atef M., Cui B., Abd El-Aty A.M. (2022). The Formation of Starch-Lipid Complexes by Microwave Heating. Food Chem..

[162] Raza H., Liang Q., Ameer K., Ma H., Ren X. (2022). Dual-Frequency Power Ultrasound Effects on the Complexing Index, Physicochemical Properties, and Digestion Mechanism of Arrowhead Starch-Lipid Complexes. Ultrason. Sonochem..

[163] Li X., Gao X., Lu J., Mao X., Wang Y., Feng D., Cao J., Huang L., Gao W. (2019). Complex Formation, Physicochemical Properties of Different Concentration of Palmitic Acid Yam (Dioscorea Pposita Thunb.) Starch Preparation Mixtures. LWT.

[164] Reddy C.K., Choi S.M., Lee D.J., Lim S.T. (2018). Complex Formation between Starch and Stearic Acid: Effect of Enzymatic Debranching for Starch. Food Chem..

[165] Chen J., Xiao J., Wang Z., Cheng H., Zhang Y., Lin B., Qin L., Bai Y. (2020). Effects of Reaction Condition on Glycosidic Linkage Structure, Physical–Chemical Properties and in Vitro Digestibility of Pyrodextrins Prepared from Native Waxy Maize Starch. Food Chem..

[166] Trithavisup K., Shi Y.C., Krusong K., Tananuwong K. (2022). Molecular Structure and Properties of Cassava-Based Resistant Maltodextrins. Food Chem..

[167] Li H., Ji J., Yang L., Lei N., Wang J., Sun B. (2020). Structural and Physicochemical Property Changes during Pyroconversion of Native Maize Starch. Carbohydr. Polym..

[168] Weil W., Weil R.C., Keawsompong S., Sriroth K., Seib P.A., Shi Y.C. (2021). Pyrodextrins from Waxy and Normal Tapioca Starches: Molecular Structure and in Vitro Digestibility. Carbohydr. Polym..

[169] Nunes F.M., Lopes E.S., Moreira A.S.P., Simões J., Coimbra M.A., Domingues R.M. (2016). Formation of Type 4 Resistant Starch and Maltodextrins from Amylose and Amylopectin upon Dry Heating: A Model Study. Carbohydr. Polym..

[170] Kapusniak K., Lubas K., Wojcik M., Rosicka-Kaczmarek J., Pavlyuk V., Kluziak K., Gonçalves I., Lopes J., Coimbra M.A., Kapusniak J. (2021). Effect of Continuous and Discontinuous Microwave-Assisted Heating on Starch-Derived Dietary Fiber Production. Molecules.

[171] Toraya-Avilés R., Segura-Campos M., Chel-Guerrero L., Betancur-Ancona D. (2017). Some Nutritional Characteristics of Enzymatically Resistant Maltodextrin from Cassava (*Manihot esculenta* Crantz) Starch. Plant Foods Hum. Nutr..

[172] Kapusniak K., Nebesny E. (2017). Enzyme-resistant dextrins from potato starch for potential application in the beverage industry. Carbohydr. Polym..

[173] Alvani K., Qi X., Tester R.F. (2011). Use of carbohydrates, including dextrins, for oral delivery. Starch Stärke.

[174] Bai Y., Shi Y.C. (2016). Chemical Structures in Pyrodextrin Determined by Nuclear Magnetic Resonance Spectroscopy. Carbohydr. Polym..

[175] Olvera-Hernández V., Betancur-Ancona D., Chel-Guerrero L.A., Ble-Castillo J.L., Castellanos-Ruelas A.F. (2018). Morphological and Physicochemical Changes in Great Dwarf Banana (*Musa cavendish* AAA) Starch Modified by Pyrodextrinization and Enzymatic Hydrolysis. Starch Stärke.

[176] Olvera Hernandez V., Ble Castillo J.L., Betancur Ancona D., Acevedo Fernandez J.J., Castellanos Ruelas A., Chel Guerrero L. (2018). Effects of modified banana (*Musa cavendish*) starch on glycemic control and blood pressure in rats with high sucrose diet. Nutr. Hosp..

[177] Ekaette I., Saldaña M.D.A. (2021). Ultrasound-Assisted Modification of Rutin to Nanocrystals and Its Application in Barley Starch Pyrodextrinization. Food Chem..

[178] de Souza Oliveira E., Lovera M., Rios Pires V., da Silva Mendes F.R., Lima Peixoto Maia N.V., Viana Rodrigues J.P., Rocha Bastos M.D., Cheng H.N., Biswas A., de Azevedo Moreira R. (2022). Effect of acid catalyst on pyroconversion of breadfruit (*Artocarpus altilis*) starch: Physicochemical and structural properties. J. Food Process Preserv..

[179] Trithavisup K., Krusong K., Tananuwong K. (2019). In-Depth Study of the Changes in Properties and Molecular Structure of Cassava Starch during Resistant Dextrin Preparation. Food Chem..

[180] Toraya-Avilés R., Segura-Campos M., Chel-Guerrero L., Betancur-Ancona D. (2017). Effects of pyroconversion and enzymatic hydrolysis on indigestible starch content and physicochemical properties of cassava (*Manihot esculenta*) starch. Starch Stärke.

[181] Lin C.L., Lin J.H., Zeng H.M., Wu Y.H., Chang Y.H. (2018). Indigestible Pyrodextrins Prepared from Corn Starch in the Presence of Glacial Acetic Acid. Carbohydr. Polym..

[182] Mao H., Li J., Chen Z., Yan S., Li H., Wen Y., Wang J. (2021). Molecular Structure of Different Prepared Pyrodextrins and the Inhibitory Effects on Starch Retrogradation. Food Res. Int..

[183] Mao H., Chen Z., Li J., Zhai X., Li H., Wen Y., Wang J., Sun B. (2021). Structural Comparisons of Pyrodextrins during Thermal Degradation Process: The Role of Hydrochloric Acid. Food Chem..

[184] Huang Z., Wang J.J., Chen Y., Wei N., Hou Y., Bai W., Hu S.Q. (2020). Effect of Water-Soluble Dietary Fiber Resistant Dextrin on Flour and Bread Qualities. Food Chem..

[185] Weil W., Weil R.C., Keawsompong S., Sriroth K., Seib P.A., Shi Y.C. (2020). Pyrodextrin from Waxy and Normal Tapioca Starches: Physicochemical Properties. Food Hydrocoll..

[186] Kapusniak (Jochym) K., Wojcik M., Wrobel K., Rosicka-Kaczmarek J., Kapusniak J. (2021). Assessment of physicochemical and thermal properties of soluble dextrin fiber from potato starch for use in fruit mousses. J. Sci. Food Agric..

[187] Kapusniak K., Ptak S., Zarski A., Nebesny E., Kapusniak J. (2014). Unconventional method for preparation of soluble fibres from starch. Agro Food Ind. Hi Tech.

[188] Kapusniak J., Kapusniak K., Ptak S., Barczynska R., Zarski A. (2014). Products of thermolysis of potato starch treated with hydrochloric and citric acids as potential prebiotics. Qual. Assur. Saf. Crop..

[189] Barczynska R., Slizewska K., Jochym K., Kapusniak J., Libudzisz Z. (2012). The Tartaric Acid-Modified Enzyme-Resistant Dextrin from Potato Starch as Potential Prebiotic. J. Funct. Foods.

[190] Slizewska K., Barczynska R., Kapusniak J., Jochym K. (2015). The Effect of Tartaric Acid-modified Enzyme-resistant Dextrin from Potato Starch on Growth and Metabolism of Intestinal Bacteria. J. Plant Pathol. Microb..

[191] Jochym K., Kapusniak J., Barczynska R., Śliżewska K. (2012). New starch preparations resistant to enzymatic digestion. J. Sci. Food Agric..

[192] Lam N.D., Quynh T.M., Diep T.B., Binh P.T., Lam T.D. (2021). Effect of gamma irradiation and pyrolysis on indigestible fraction, physicochemical properties, and molecular structure of rice starch. J. Food Process Preserv..

[193] Gómez Pamies L.C., Lataza Rovaletti M.M., Martinez Amezaga N.M.J., Benítez E.I. (2021). The Impact of Pirodextrin Addition to Improve Physicochemical Parameters of Sorghum Beer. LWT.

[194] Bai Y., Cai L., Doutch J., Gilbert E.P., Shi Y.-C. (2014). Structural Changes from Native Waxy Maize Starch Granules to Cold-Water-Soluble Pyrodextrin during Thermal Treatment. J. Agric. Food Chem..

[195] Cao Y., Chen X., Sun Y., Shi J., Xu X., Shi Y.-C. (2018). Hypoglycemic Effects of Pyrodextrins with Different Molecular Weights and Digestibilities in Mice with Diet-Induced Obesity. J. Agric. Food Chem..

[196] Han X., Kang J., Bai Y., Xue M., Shi Y.C. (2018). Structure of Pyrodextrin in Relation to Its Retrogradation Properties. Food Chem..

[197] Sun Z., Kang J., Shi Y.C. (2021). Changes in Molecular Size and Shape of Waxy Maize Starch during Dextrinization. Food Chem..

[198] Sun Z., Shi J., Shi Y.C. (2022). Dissolution of Waxy Maize Pyrodextrin Granules in Mixtures of Glycerol and Water, Separating Loss of Crystallinity from Loss of Birefringence. Carbohydr. Polym..

[199] Lovera M., de Castro G.M.C., da Rocha Pires N., Bastos M.D.S.R., Holanda-Araújo M.L., Laurentin A., de Azevedo Moreira R., de Oliveira H.D. (2020). Pyrodextrinization of Yam (*Dioscorea* Sp.) Starch Isolated from Tubers Grown in Brazil and Physicochemical Characterization of Yellow Pyrodextrins. Carbohydr. Polym..

[200] Udomrati S., Ikeda S., Gohtani S. (2011). The effect of tapioca maltodextrins on the stability of oil-in-water emulsions. Starke.

[201] Klinkesorn U., Sophanodora P., Chinachoti P., McClements D. (2004). Stability and rheology of corn oil-in-water emulsions containing maltodextrin. Food Res. Int..

[202] D’hulst A., Verkebe N. (1992). Chiral separation by capillary electrophoresis with oligosaccharides. J. Chromatogr. A..

[203] Sun P., Yang H.J., Wang Y.-Q., Liu K.-Z., Xu Y.-W. (2013). Lipase-catalyzed synthesis and characterization of stearic acid dextrin ester. Res. Health Nutr..

[204] Dokic P., Jakovljenic J., Dokic-Baucal L. (1998). Molecular characteristics of maltodextrins and rheological behaviour of diluted and concentrated solutions. Colloids Surf. A Physicochem. Eng. Asp..

[205] Dokic-Baucal L., Dokic P., Jakovljevic J. (2004). Influence of different maltodextrins on properties of O/W emulsions. Food Hydrocoll..

[206] Biswas A., Shogren R., Willet J. (2009). Ionic liquid as a solvent and catalyst for acylation of maltodextrin. Ind. Crops Prod..

[207] Udomrati S., Gohtani S. (2014). Enzymatic esterification of tapioca maltodextrin fatty acid ester. Carbohydr. Polym..

[208] Udomrati S., Khalid N., Gohtani S., Nakajima M., Neves M.A., Uemura K., Kobayashi I. (2016). Effect of esterified oligosaccharides on the formation and stability of oil-in-water emulsions. Carbohydr. Polym..

[209] Dickinson E. (2003). Hydrocolloids at interfaces and the influence on the properties of dispersed systems. Food Hydrocoll..

[210] Schirle-Keller J.P.X. (1996). Flavour interaction with fat replacers and aspartame. Diss. Abstr. Int. B.

[211] Sadtler V.M., Imbert P., Dellacherie E. (2002). Ostwald ripening of oil-in-water emulsions stabilized by phenoxy-substituted dextrans. J. Colloid Interface Sci..

[212] van den Broek L.A.M., Boeriu C.G. (2013). Enzymatic synthesis of oligo- and polysaccharide fatty acid esters. Carbohydr. Polym..

[213] Kaewprapan K., Baros F., Maie E., Inprakhon P., Durand A. (2012). Macromolecular surfactants synthesized by lipase-catalyzed transesterification of dextran with vinyl decanoate. Carbohydr. Polym..

[214] Neta N.S., Teixeira J.A., Rodrigues L.R. (2014). Sugar Ester Surfactants: Enzymatic Synthesis and Applications in Food Industry. Crit. Rev. Food Sci. Nutr..

[215] Chang S.W., Shaw J.F. (2009). Biocatalysis for the production of carbohydrate esters. New Biotechnol..

[216] Udomrati S., Gohtani S. (2015). Tapioca maltodextrin fatty acid ester as a potential stabilizer for Tween 80-stabilized oil-in-water emulsions. Food Hydrocoll..

[217] Udomrati S., Nopparat C., Gohtani S., Vipa S., Supakchon K. (2020). Emulsion stabilization mechanism of combination of esterified maltodextrin and Tween 80 in oil-in-water emulsions. Food Sci. Biotechnol..

[218] Roczkowska M., Ptak S., Siemion P., Kapusniak J. Biocatalysed esterification of potato maltodextrin in the presence of fungal lipase. Proceedings of the 11th International Conference on Polysaccharides-Glycoscience, Czech Chemical Society, Novotneho Lavka 5.

[219] Roczkowska M., Kapusniak J. Modified maltodextrins as ecological surfactants for food purposes. Proceedings of the XLIII Scientific Session of the Committee on Food and Nutrition Sciences of the Polish Academy of Sciences Food for the Future, Publishing House of the Wroclaw University of Environmental and Life Sciences.

[220] Park N., Walsh M. (2019). Physical and emulsion stabilizing properties of maltodextrin fatty acid polymers produced by lipase-catalyzed reactions in ethanol. Carbohydr. Polym..

[221] Pantoa T., Shompoosang S., Ploypetchara T., Gohtani S., Udomrati S. (2019). Surface-active properties and anti-microbial activities of esterified maltodextrins. Starch Stärke.

[222] Park N., Walsh M. (2021). Microbial inhibitory properties of maltodextrin fatty acid esters against food-related microorganisms. LWT Food Sci. Technol..

[223] Zhao L., Zhang H., Hao T., Li S. (2015). In vitro antibacterial activities and mechanism of sugar fatty acid esters against five food-related bacteria. Food Chem..

[224] Ma Y., Hou C.J., Wu H.X., Fa H.B., Li J.J., Shen C.H., Li D., Huo D.Q. (2016). Synthesis of maltodextrin-grafted-cinnamic acid and evaluation on its ability to stabilize anthocyanins via microencapsulation. J. Microencapsul..

[225] Ma Y., Hou C.J., Fa H.B., Huo D.Q., Yang M. (2016). Synthesis and antioxidant property of hydroxycinnamoyl maltodextrin derivatives. Int. J. Food Sci. Technol..

[226] Wang S., Wang Q., Fan X., Xu J., Zhang Y., Yuan J., Jin H., Cavaco-Paulo A. (2016). Synthesis and characterization of starch-poly (methyl acrylate) graft copolymers using horseradish peroxidase. Carbohydr. Polym..

[227] Wang Y., Xin J., Shi J., Wu W., Xia C. (2014). A kinetic study of starch palmitate synthesis by immobilized lipase-catalyzed esterification in solvent free system. J. Mol. Catal. B Enzym..

[228] Xin J.Y., Wang Y., Liu T., Lin K., Chang L., Xia C.G. (2012). Biosysthesis of corn starch palmitate by lipase Novozym 435. Int. J. Mol. Sci..

[229] Lin R., Li H., Long H., Su J., Huang W., Wang S. (2014). Optimization of lipase-catalyzed rosin acid starch synthesis by response surface methodology. J. Mol. Catal. B Enzym..

[230] Varavinit S., Chaokasem N., Shobsngob S. (2001). Studies of flavor encapsulation by agents produced from modified sago and tapioca starches. Starch Stärke.

[231] Lu X., Luo Z., Yu S., Fu X. (2012). Lipase-catalyzed synthesis of starch palmitate in mixed ionic liquids. J. Agric. Food Chem..

[232] Lin R., Li H., Long H., Su J., Huang W. (2015). Structure and characteristics of lipase-catalyzed rosin acid starch. Food Hydrocoll..

[233] Xu J., Zhou C.W., Wang R.Z., Yang L., Du S.S., Wang F.P., Ruan H., He G.Q. (2012). Lipase-coupling esterification of starch with octenyl succinic anhydride. Carbohydr. Polym..

[234] Lu K., Miao M., Ye F., Cui S.W., Li X., Jiang B. (2016). Impact of dual-enzyme treatment on the octenylsuccinic anhydride esterification of soluble starch nanoparticle. Carbohydr. Polym..

[235] Gao Y., Wang L., Yue X., Xiong G., Wu W., Qiao Y., Liao L. (2014). Physicochemical properties of lipase-catalyzed laurylation of corn starch. Starch Stärke.

[236] Zarski A., Ptak S., Siemion P., Kapusniak J. (2016). Esterification of potato starch by a biocatalysed reaction in an ionic liquid. Carbohydr. Polym..

[237] Zarski A., Bajer K., Zarska S., Kapusniak J. (2019). From high oleic vegetable oils to hydrophobic starch derivatives: I. Development and structural studies. Carbohydr. Polym..

[238] Zarski A., Bajer K., Raszkowska-Kaczor A., Rogacz D., Zarska S., Kapusniak J. (2020). From high oleic vegetable oils to hydrophobic starch derivatives: II. Physicochemical, processing and environmental properties. Carbohydr. Polym..

[239] Lu K., Zhu J., Bao X., Liu H., Yu L., Chen L. (2020). Effect of starch microstructure on microwave-assisted esterification. Int. J. Biol. Macromol..

[240] Yang Q., Qi L., Luo Z., Kong X., Xiao Z., Wang P., Peng X. (2017). Effect of microwave irradiation on internal molecular structure and physical properties of waxy maize starch. Food Hydrocoll..

[241] Oyeyinka S.A., Akintayo O.A., Adebo O.A., Kayitesi E., Njobeh P.B. (2021). A review on the physicochemical properties of starches modified by microwave alone and in combination with other methods. Int. J. Biol. Macromol..

[242] Zhao K., Li B., Xu M., Jing L., Gou M., Yu Z., Zheng J., Li W. (2018). Microwave pretreated esterification improved the substitution degree, structural and physicochemical properties of potato starch esters. LWT Food Sci. Technol..

[243] Lin D., Zhou W., He Q., Xing B., Wu Z., Chen H., Wu D., Zhang Q., Qin W. (2019). Study on preparation and physicochemical properties of hydroxypropylated starch with different degree of substitution under microwave assistance. Int. J. Biol. Macromol..

[244] Liu J., Ming J., Li W., Zhao G. (2012). Synthesis, characterisation and in vitro digestibility of carboxymethyl potato starch rapidly prepared with microwave-assistance. Food Chem..

[245] Hu A., Chen X., Wang J., Wang H., Zheng J., Wang L. (2021). Effects on the structure and properties of native corn starch modified by enzymatic debranching (ED), microwave assisted esterification with citric acid (MCAE) and by the dual ED/MCAE treatment. Int. J. Biol. Macromol..

[246] Horchani H., Chaabouni M., Gargouri Y., Sayari A. (2010). Solvent-free lipase-catalyzed synthesis of long-chain starch esters using microwave heating: Optimization by response surface methodology. Carbohydr. Polym..

[247] Adak S., Banerjee R. (2016). A green approach for starch modification: Esterification by lipase and novel imidazolium surfactant. Carbohydr. Polym..

[248] Lukasiewicz M., Kowalski S. (2012). Low power microwave-assisted enzymatic esterification of starch. Starch Stärke.

[249] Li X., He Y., Huang C., Zhu J., Lin A.H.M., Chen L., Li L. (2016). Inhibition of plasticizer migration from packaging to foods during microwave heating by controlling the esterified starch film structure. Food Control.

[250] Hoover R., Hughes T., Chung H., Liu Q. (2010). Composition, molecular structure, properties, and modification of pulse starches: A review. Food Res. Int..

[251] Chong W.T., Uthumporn U., Karim A.A., Cheng L.H. (2013). The influence of ultrasound on the degree of oxidation of hypochlorite-oxidized corn starch. LWT Food Sci. Technol..

[252] Apopei Loghin D.F., Biliuta G., Coseri S., Dragan E.S. (2017). Preparation and characterization of oxidized starch/poly(N,N-dimethylaminoethyl methacrylate) semi-IPN cryogels and in vitro controlled release evaluation of indomethacin. Int. J. Biol. Macromol..

[253] Biliuta G., Sacarescu L., Socoliuc V., Iacob M., Gheorghe L., Negru D., Coseri S. (2017). Carboxylated Polysaccharides Decorated with Ultrasmall Magnetic Nanoparticles with Antibacterial and MRI Properties. Macromol. Chem. Phys..

[254] Baron R.I., Bercea M., Avadanei M., Lisa G., Biliuta G., Coseri S. (2019). Green route to produce self-healable hydrogels based on tricarboxy cellulose and poly (vinyl alcohol). Int. J. Biol. Macromol..

[255] Culica M.E., Avadanei M., Baron R.I., Chibac-Scutaru A.L., Asandulesa M., Biliuta G., Lisa G., Coseri S. (2020). The source of conductivity and proton dynamics study in TEMPO-oxidized cellulose doped with various heterocyclic molecules. Cellulose.

[256] Baron R.I., Coseri S. (2020). Preparation of water-soluble cellulose derivatives using TEMPO radical-mediated oxidation at extended reaction time. React. Funct. Polym..

[257] Duceac I.A., Vereștiuc L., Coroaba A., Arotăriței D., Coseri S. (2021). All-polysaccharide hydrogels for drug delivery applications: Tunable chitosan beads surfaces via physical or chemical interactions, using oxidized pullulans. Int. J. Biol. Macromol..

[258] Bercea M., Biliuta G., Avadanei M., Baron R.I., Butnaru M., Coseri S. (2019). Self-healing hydrogels of oxidized pullulan and poly(vinyl alcohol). Carbohydr. Polym..

[259] Baron R.I., Culica M.E., Biliuta G., Bercea M., Gherman S., Zavastin D., Ochiuz L., Avadanei M., Coseri S. (2019). Physical Hydrogels of Oxidized Polysaccharides and Poly(Vinyl Alcohol) for Wound Dressing Applications. Materials.

[260] Hao J., Wu F., Tang R., Sunb Y., Liu D., Zhang Z. (2020). Preparation of 1,4-linked α-D-glucuronans from starch with 4-acetamide-TEMPO/NaClO_2_/NaClO system. Int. J. Biol. Macromol..

[261] Yang G., Xia Y., Lin Z., Zhang K., Fatehi P., Chen J. (2021). Physicochemical impact of cellulose nanocrystal on oxidation of starch and starch based composite films. Int. J. Biol. Macromol..

[262] Zuo Y., Liu W., Xiao J., Zhao X., Zhu Y., Wu Y. (2017). Preparation and characterization of dialdehyde starch by one-step acid hydrolysis and oxidation. Int. J. Biol. Macromol..

[263] Dou Y., Huang X., Zhang B., He M., Yin G., Cui Y. (2015). Preparation and characterization of a dialdehyde starch crosslinked feather keratin film for food packaging application. RSC Adv..

[264] Wu W., Li Y., Yang L., Ma Y., Pan D., Li Y. (2014). A Facile One-Pot Preparation of Dialdehyde Starch Reduced Graphene Oxide/Polyaniline Composite for Supercapacitors. Electrochim. Acta.

[265] Lewicka K., Siemion P., Kurcok P. (2015). Chemical Modifications of Starch: Microwave Effect. Int. J. Polym. Sci..

[266] Lukasiewicz M., Bednarz S., Ptaszek A. (2011). Environmental friendly polysaccharide modification—Microwave-assisted oxidation of starch. Starch Stärke.

[267] León O., Soto D., López D., Muñoz-Bonilla A., Fernández-García M. (2019). Fat-Replacer Properties of Oxidized Cassava Starch Using Hydrogen Peroxide/Sodium Bicarbonate Redox System in Mayonnaise Formulation and Its Stability. Starch Stärke.

[268] Li J., Zhou M., Cheng F., Lin Y., Shi L., Zhu P.-X. (2020). Preparation of oxidized corn starch with high degree of oxidation by fenton-like oxidation assisted with ball milling. Mater. Today Commun..

[269] Barbosa J.V., Martins J., Carvalho L., Bastos M.M.S.M., Magalhães F.D. (2019). Effect of peroxide oxidation on the expansion of potato starch foam. Ind Crops Prod..

[270] Maache-Rezzoug Z., Zarguili I., Loisel C., Queveau D., Buléon A. (2008). Structural modifications and thermal transitions of standard maize starch after DIC hydrothermal treatment. Carbohydr. Polym..

[271] Bahrani S.A., Loisel C., Rezzoug S.A., Doublier J.L., Maache-Rezzoug Z. (2012). Role of vacuum steps added before and after steaming treatment of maize starch. Impact on pasting, morphological and rheological properties. Carbohydr. Polym..

[272] Zhang Q., Zhang S., Deng L., Wu C., Zhong G. (2018). Effect of Vacuum Treatment on the Characteristics of Oxidized Starches Prepared Using a Green Method. Starch Stärke.

[273] Wei B., Qi H., Wang Z., Bi Y., Zou J., Xu B., Ren X., Ma H. (2020). The *ex-situ* and *in-situ* ultrasonic assisted oxidation of corn starch: A comparative study. Ultrason. Sonochem..

[274] Fan Z. (2015). Impact of ultrasound on structure, physicochemical properties, modifications, and applications of starch. Trends Food Sci. Technol..

[275] Tian Y.L., Ying M., Shi F.Y., Shi J., Xue S. (2011). The effect of ball milling treatment on structure and porosity of maize starch granule. Innov. Food Sci. Emerg. Technol..

[276] Zhang J., Cao S., Liu P., Shan Z. (2022). Electrochemical Oxidation of Starch Investigated by Single-Current-Transition Method. Starch Stärke.

[277] Sevket T., Melayib B. (2019). Enhancement of anaerobic digestion of waste activated sludge by chemical pretreatment. Fuel.

[278] Hornung P.S., Avila S., Lazzarotto M., da Silveira Lazzarotto S.R., de Andrade de Siqueira G.L., Schnitzler E., Ribani R.H. (2017). Enhancement of the functional properties of *Dioscoreaceas* native starches: Mixture as a green modification process. Thermochim. Acta.

[279] Dang X., Chen H., Wang Y., Shan Z. (2018). Freeze-drying of oxidized corn starch: Electrochemical synthesis and characterization. Cellulose.

[280] Kurdziel M., Łabanowska M., Pietrzyk S., Sobolewska-Zielińska J., Michalec M. (2019). Changes in the physicochemical properties of barley and oat starches upon the use of environmentally friendly oxidation methods. Carbohydr. Polym..

[281] Çatal H., Ibanoglu S. (2014). Effect of aqueous ozonation on the pasting, flow and gelatinization properties of wheat starch. Food Sci. Technol..

[282] Castanha N., Matta M.D., Augusto P.E.D. (2017). Potato starch modification using the ozone technology. Food Hydrocoll..

